# The Epigenetic Landscape of Latent Kaposi Sarcoma-Associated Herpesvirus Genomes

**DOI:** 10.1371/journal.ppat.1000935

**Published:** 2010-06-03

**Authors:** Thomas Günther, Adam Grundhoff

**Affiliations:** Heinrich-Pette-Institute for Experimental Virology and Immunology, Hamburg, Germany; Sanger Institute, United Kingdom

## Abstract

Herpesvirus latency is generally thought to be governed by epigenetic modifications, but the dynamics of viral chromatin at early timepoints of latent infection are poorly understood. Here, we report a comprehensive spatial and temporal analysis of DNA methylation and histone modifications during latent infection with Kaposi Sarcoma-associated herpesvirus (KSHV), the etiologic agent of Kaposi Sarcoma and primary effusion lymphoma (PEL). By use of high resolution tiling microarrays in conjunction with immunoprecipitation of methylated DNA (MeDIP) or modified histones (chromatin IP, ChIP), our study revealed highly distinct landscapes of epigenetic modifications associated with latent KSHV infection in several tumor-derived cell lines as well as *de novo* infected endothelial cells. We find that KSHV genomes are subject to profound methylation at CpG dinucleotides, leading to the establishment of characteristic global DNA methylation patterns. However, such patterns evolve slowly and thus are unlikely to control early latency. In contrast, we observed that latency-specific histone modification patterns were rapidly established upon a *de novo* infection. Our analysis furthermore demonstrates that such patterns are not characterized by the absence of activating histone modifications, as H3K9/K14-ac and H3K4-me3 marks were prominently detected at several loci, including the promoter of the lytic cycle transactivator Rta. While these regions were furthermore largely devoid of the constitutive heterochromatin marker H3K9-me3, we observed rapid and widespread deposition of H3K27-me3 across latent KSHV genomes, a bivalent modification which is able to repress transcription in spite of the simultaneous presence of activating marks. Our findings suggest that the modification patterns identified here induce a poised state of repression during viral latency, which can be rapidly reversed once the lytic cycle is induced.

## Author Summary

A characteristic feature of herpesviruses is their ability to establish a latent infection during which most of the viral genes are silenced. As a consequence, no viral progeny is produced and the host cell remains viable. While the viral genome may persist in the nucleus of such cells indefinitely, it retains the ability to re-enter the lytic cycle and produce new virions if conditions in the cell become unfavorable. The molecular requirements for the establishment of latency are poorly understood, but are thought to depend on epigenetic modifications of the viral episome. Here, we report a genome-wide screen to investigate DNA methylation and histone modification patterns associated with latent infection by Kaposi Sarcoma-associated herpesvirus (KSHV), a tumor virus linked to the development of several cancers. We find that latency is likely to be determined by modifications commonly associated with genes that are transcriptionally “poised”. The promoters of such genes harbor activating as well as repressive histone marks such that they are silenced, but they can be rapidly activated upon removal of the repressive marks. Our findings thus may explain how KSHV achieves efficient quiescence during latency, yet retains the potential to quickly revert to a fully active state upon induction of the lytic cycle.

## Introduction

Herpesviruses are able to establish latent infections, enabling them to persist for the lifetime of their host [1]. During latency, no viral progeny is produced; instead, the largely quiescent genome persists as an extrachromosomal episome in the nucleus of the infected cell. Unfavorable conditions (e.g. cell stress) may trigger reactivation of such cells, leading to induction of the lytic cycle and completion of the viral lifecycle. In a healthy host, latently infected cells form a reservoir of chronic viral infection which is tightly controlled by the immune system. However, latently infected cells may also give rise to disease if the immunological control is lost. This is especially true for the members of the gammaherpesvirus subfamily, which are frequently associated with tumors in their natural host, in particular in immunosuppressed individuals. Kaposi Sarcoma-associated herpesvirus (KSHV) is etiologically linked to Kaposi Sarcoma (KS), a tumor of endothelial origin, as well as at least two lymphoproliferative disorders, primary effusion lymphoma (PEL) and multicentric Castleman's disease (MCD) [2], [3], [4]. The majority of tumor cells in these malignancies exhibit a latent gene expression profile which has been extensively studied in cell lines established from PEL tumors [5], [6], [7]. These cells express a very limited contingent of viral genes, including the latency-associated nuclear antigen LANA (encoded by ORF73) which permits replication of latent episomes, a viral cyclin D homologue (v-Cyc/ORF72), a viral homologue of a FLICE-inhibitory protein (v-Flip) encoded by ORF71 (also termed K13) and Kaposin (ORF K12), a protein that can stabilize cytokine transcripts [7], [8], [9], [10], [11], [12]. All of the above proteins are translated from alternatively spliced mRNAs transcribed from a single multicistronic locus; primary transcripts from the locus furthermore can give rise to 12 virally encoded microRNAs (miRNAs) [7], [13], [14], [15], [16], [17], [18]. It is thought that, together, these genes serve to ensure persistence of the latent infection and survival of the host cell. However, several of the latency genes have also been shown to exhibit tumorigenic properties in various experimental systems, supporting the idea that the viral latency program plays a causative role during onset and/or progression of KSHV associated tumors.

The viral genes which encode components of the lytic or productive cycle are transcriptionally silent during latency. This quiescent state of infection can be overturned by forced expression of Rta (the product of the ORF50 gene, also termed Lyta), a homologue of the Epstein-Barr virus (EBV) transactivator Rta [19], [20], [21]. Upon expression, Rta acts as a master-switch regulator which orchestrates the expression of downstream lytic genes, leading to massive amplification of viral genomes, followed by assembly of virions and, ultimately, death of the host cells and release of viral progeny [20], [21], [22], [23]. How Rta and other lytic genes are kept silenced during latency is not understood, but it is very likely that epigenetic modifications play an important role during this process. This notion is supported by the fact that treatment of latently infected PEL cells with inhibitors of DNA methyltransferases as well as histone deacetylases induces lytic cycle replication, and that lytic cycle induction leads to profound chromatin rearrangements at several loci [24], [25], [26], [27]. Furthermore, the ORF50 promoter was reported to be subject to DNA methylation in latently infected PEL cells whereas the latent ORF73 promoter remained unmethylated, and it has therefore been suggested that CpG methylation may actively repress Rta expression during latency [26]. The DNA methylation status of other regions of the KSHV genome, however, has so far not been analyzed. Likewise, the current knowledge about global histone modification patterns during viral latency is very limited. All studies of latent modification patterns have furthermore been performed in PEL cells and thus describe the epigenetic status during fully established latency. However, since the packaged virion DNA is devoid of DNA methylation as well as histones [28] and thus epigenetically naïve, such epigenetic modification patterns need to be re-established during each round of latent infection. Especially the early phase of a *de novo* infection thus represents a critical phase of the viral lifecycle. We have performed a comprehensive study of DNA methylation as well as histone modification patterns across the complete KSHV genome, in both PEL cells as well as a *de novo* infected endothelial cell line. We have observed highly distinct global patterns on the level of both DNA as well as histone modifications. Such patterns were furthermore highly similar in PEL cells and stably infected endothelial cells, suggesting a highly regulated modification program during latency establishment. However, whereas modified histones could be readily detected at early timepoints of a *de novo* infection, DNA methylation patterns evolved over significantly longer periods of time, suggesting they do not govern early latency expression patterns. Our analysis rather suggests that DNA methylation patterns evolve as a secondary result of the histone modification patterns, which are established early in the infection. Surprisingly, in spite of their quiescent state, latent KSHV episomes were also not devoid of activating histone marks: In fact, such marks occupied several lytic promoters soon after the *de novo* infection and were not stripped from the genomes in long term infected cells. However, concomitant with the appearance of these modifications, latent genomes were also subject to profound tri-methylation of lysine 27 of histone H3 (H3K27), a modification which can suppress transcription even in the presence of activating marks [29], [30], [31]. Thus, latent episomes bear the hallmarks of poised chromatin, an observation which is in line with the hypothesis that viral latency represents a meta-stable state of transcriptional repression which can be quickly reversed once the lytic cycle is induced.

## Results/Discussion

### DNA methylation patterns of latent KSHV genomes

In mammals, DNA methylation occurs almost exclusively by methylation of cytidine residues at CpG dinucleotides and is generally associated with transcriptional repression (reviewed in [32]). As methylcytidine is prone to spontaneous deamination, an evolutionary consequence of DNA methylation is the relative scarcity of CpG dinucleotides in methylated genomes. In contrast to most members of the alpha- and betaherpesvirus subfamily, the majority of gammaherpesviruses show evidence of such CpG suppression, suggesting that these viruses are subject to DNA methylation [26]. Furthermore, the genomes of EBV as well as the rhadinovirus Herpesvirus saimiri (HVS) have been found to carry methylated CpG motifs at multiple loci in latently infected cells, suggesting that DNA methylation plays a role in the control of latent gammaherpesvirus gene expression patterns (reviewed in [33], [34]). So far, analysis of DNA methylation within latent KSHV genomes has been limited to the promoters of the gene encoding Rta (i.e. ORF50) and the promoter upstream of ORF73/LANA which drives expression of the latency gene cluster. While no CpG methylation was detected in the region of the ORF73 promoter, the ORF50 promoter was found to be heavily methylated in the PEL derived cell line BCBL-1 [26]. As promoter activity was furthermore repressed by DNA methylation in an *in vitro* assay, it was suggested that CpG methylation actively suppresses expression of the lytic switch gene Rta during KSHV latency [26]. However, this hypothesis is complicated by the fact that the same study also found that the majority of samples from different KSHV-positive tumor samples did not harbor these methylation patterns. The authors suggest that their observations may have been due to the presence of lytic cells, which represent a sub-population among the mostly latently infected cells in some tumor types.

**Figure 1:**
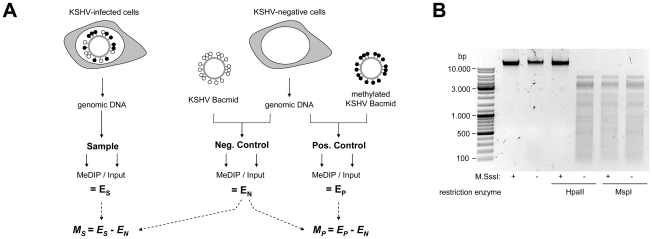
Experimental design of MeDIP analysis. **A**: Schematic representation of the experimental setup for the analysis of CpG methylation patterns. The KSHV episome in infected cells is expected to be partially methylated, as indicated by black and white circles which symbolize methylated or unmethylated CpG dinucleotides, respectively. Genomic DNA was isolated from such cells and the samples were subjected to immunoprecipitation using a methylcytidine specific antibody (MeDIP procedure), followed by hybridization of the precipitated samples versus the input on tiling microarrays. For each probe, an enrichment score E_S_ was calculated, which represents the ratio of MeDIP over input fluorescence signals. The efficiency of the immunoprecipitation depends on the total number of methylated CpG motifs in a given fragment and E_S_ is thus a function of the extend of methylation as well as local CpG frequencies. Therefore, to obtain reference values which signify maximum methylation for each probe, we generated a positive control by subjecting KSHV bacmids to CpG methylation *in vitro*. The bacmid was mixed with cellular DNA to simulate the host background and subjected to the same MeDIP procedure as samples from infected cells. Similarly, a negative control of unmethylated bacmid was prepared to control for cross-hybridization of unspecific background. After normalization of the array data using a spike-in control (see Material & Methods for details), background-corrected methylation values M_S_ and M_P_ were calculated for each probe by subtraction of the corresponding negative control value. **B**: Confirmation of successful *in vitro* methylation of KSHV bacmids used as a positive control. A bacmid carrying the complete KSHV genome (BAC36 [36]) was methylated using M.SssI, a methyltransferase specific for CpG dinucleotides. Methylated or unmethylated bacmids were subjected to restriction digestion using the methylation sensitive enzyme HpaII and its isoschizomer MspI, which cuts regardless of methylation. Methylated bacmids were resistant to HpaII digestion, signifying complete methylation.

Given the absence of comprehensive DNA methylation data for KSHV, we first sought to determine the global methylation status of viral episomes in PEL derived cell lines. For this purpose, we employed the MeDIP (methylated DNA immunoprecipitation) technique, which is based on the pulldown of methylated DNA using methylcytidine-specific antibodies [35]. The MeDIP samples were analyzed on a custom-designed, high-resolution microarray which covers both strands of the KSHV genome in non-overlapping, hybridization temperature-optimized 60mers. To obtain a quantitative measure of the extent of DNA methylation, we additionally devised positive and negative controls according to the scheme depicted in Figure 1A. As a negative control, we employed a bacterially amplified (and hence CpG methylation free) bacmid clone which carries the complete KSHV genome [36]. For a given DNA fragment, the amount of DNA which can be maximally recovered by MeDIP is dependent on the number of CpG motifs in that sequence. Consequently, the array hybridization patterns are a function of the relative degree of methylation as well as local CpG frequency. To control for such differences we generated a positive control by *in vitro* methylation of KSHV bacmids using the methylase M. SssI, which is specific for CpG dinucleotides. Restriction analysis confirmed complete methylation of the bacmid DNA (Fig. 1B). Prior to immunoprecipitation, the untreated or *in vitro* methylated bacmid DNA was mixed with DNA from human cell lines to also control for any signals which may arise due to cross-hybridization of cellular DNA in the infected samples. The ratio of viral and cellular DNA was selected such that it is equal to that typically seen in KSHV-infected PEL cell lines and corresponds to a viral copy number of approximately 30 genomes per cell. Furthermore, all samples were spiked with a constant amount of *in vitro* methylated heterologous DNA, so that accurate normalization across individual array hybridizations could be performed. After normalization, the MeDIP values obtained from the samples or the positive control were corrected by subtracting the background values from the negative control (see Material & Methods for details).

**Figure 2:**
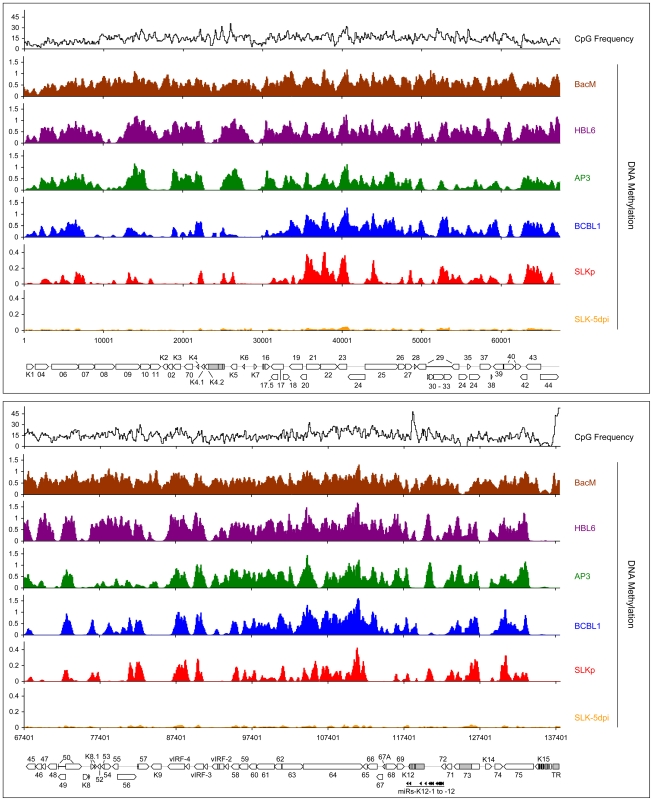
Global DNA methylation patterns of latent KSHV genomes. Global DNA methylation patterns of KSHV genomes in PEL cells (HBL6, AP3 and BCBL1), long-term *in vitro* infected endothelial SLK cells (SLKp) or SLK cultures 5 days after *de novo* infection with KSHV (SLK-5dpi) were determined by MeDIP array analysis as described in the text. The profile observed for the positive control, consisting of a completely methylated KSHV bacmid mixed with cellular DNA, is also shown (BacM). CpG methylation values are shown on the y-axis for overlapping 250 bp sequence windows, shifted along the KSHV genome in increments of 100 bp. Methylation values of individual windows represent the mean of background-corrected methylation values from all probes matching either strand of the window (see Material & Methods for details). The number of CpG dinucleotides which are present in each sequence window are shown at the top. The nucleotide positions and genome map shown at the bottom of each panel refer to the reference KSHV sequence (NC_009333). Open reading frames and repeat regions are indicated as block arrows and grey boxes, respectively.

To minimize the risk of investigating cell-line specific (and hence potentially random) modifications, we analyzed the global DNA methylation pattern of KSHV genomes in three different PEL-derived cell lines: BCBL1, AP3 and HBL6. The HBL6 line was originally established from a PEL tumor co-infected with KSHV and EBV and carries both viruses in a latent state [37]. BCBL1 and AP3 cells are KSHV positive, but negative for EBV. In Figure 2, we present the results of our analysis of PEL cells, along with the data obtained from the positive control (upper four solid graphs in each panel; see also Figure S1 for a more detailed view with a differentially scaled x-axis). The distribution of local CpG frequencies across the KSHV genome is also shown (black line graph). As expected, the positive control yielded a signal distribution which showed a high degree of correlation with local CpG content (Pearson correlation coefficient = 0.513, see Table 1). The results from the PEL-derived cell lines revealed that, indeed, KSHV genomes are subject to profound DNA methylation during latency. For all three lines, we observed global methylation profiles which were strikingly similar, with overall correlation coefficients ranging from 0.593 to 0.724 (Table 1). Furthermore, the profiles were clearly not a mere function of CpG content, as several regions showed low levels of DNA methylation in all three PEL lines, but not the positive control. One such region, extending approximately from nucleotides (nts.) 127301 to 128901, harbors the major latency promoter upstream of ORF73. The absence of methylation in this area is to be expected (and has been noted before [26]), given the constitutive activity of the promoter in latently infected cells. However, our analysis revealed several additional loci which were not (or only poorly) methylated, despite their (presumable) transcriptional inactivity in latently infected cells. For example, the region between nts. 9701 and 12601 showed very little methylation in PEL lines compared to the positive control; this area is centered on the start position of the gene encoding the DNA polymerase (ORF9), which is exclusively expressed during the lytic cycle. While the methylation profiles of the three PEL lines were highly similar, the absolute degree of methylation was different: Across the complete KSHV genome, HBL6 reached approximately 88% of the MeDIP signal obtained for the positive control, followed by AP3 (54%) and BCBL1 (51%).

**Table 1:** Pearson correlation coefficients of DNA methylation patterns.

		*MeDIP*				
	*CpG Frequency*	*BacM*	*BCBL1*	*AP3*	*HBL6*	*SLKp*
***MeDIP***						
*BacM*	0.513					
*BCBL1*	0.324	0.427				
*AP3*	0.263	0.407	0.608			
*HBL6*	0.369	0.591	0.593	0.724		
*SLKp*	0.235	0.300	0.712	0.403	0.433	
*SLK-5dpi*	0.297	0.092	0.549	0.334	0.266	0.653

*Note: correlation coefficients were calculated according to Pearson from the data shown in* *Figure 2* *. All data points are given in Dataset S1.*

To investigate whether the observed methylation profiles were specific to PEL cell lines or a general feature of latent genomes, we next sought to analyze genomes from cells which had established stable latency after KSHV infection *in vitro*. While the infection of non-adherent cells (including B cells) with KSHV *in vitro* is very inefficient, a wide variety of adherent cells can be readily infected by incubating the cultures with supernatants from lytically induced PEL lines [38]. However, although KSHV rapidly adopts a latent expression profile in these cultures, most infected cells tend to loose the viral episomes over the following cell divisions [39]. Only a small percentage of cells ultimately succeeds in establishing stable latent episomes, which are then propagated with the same efficiency as the genomes in PEL cells. Previously, we have established the SLKp sub-line from *in vitro* infected SLK cells, a cell line of endothelial origin [39]. The SLKp line was generated by pooling seven KSHV-positive single cell clones which had been isolated from an infected bulk cultures at approximately 65 days post infection. SLKp cells are stably infected, carry approximately the same episome copy number as BCBL1 cells (30–40 copies/cell), and have a strictly latent expression profile [39]. We analyzed SLKp cells which had been in continuous culture for 6 months, corresponding to a total time span of approximately 8 months after the original infection. As shown in Figure 2 (red graph), although the overall methylation levels in SLKp cells were substantially lower (reaching approximately 9.6% of positive control levels; note the differentially scaled y-axis in Figure 2), the observed profile was indeed highly similar to that seen in PEL cells, with the highest degree of similarity to BCBL1 cells (correlation coefficient 0.712, see Table 1). Taken together, these results suggest that the distinct MeDIP profiles revealed during our analysis are non-random and represent a characteristic of latent KSHV episomes.

**Figure 3 ppat-1000935-g003:**
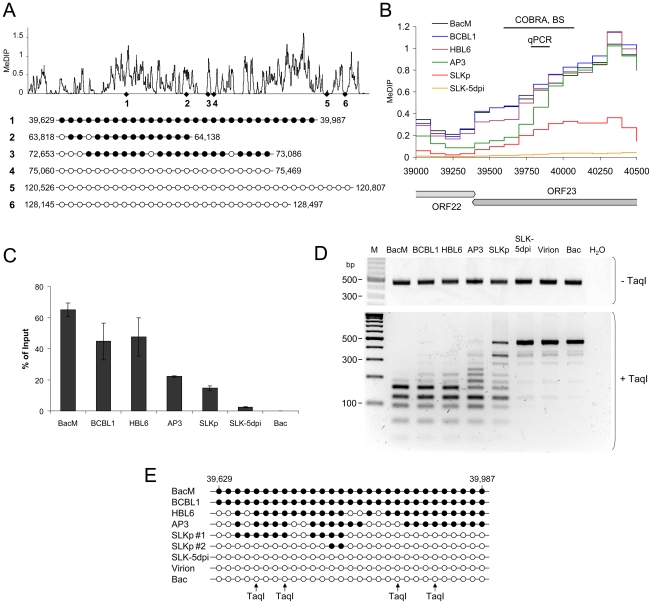
Verification of MeDIP microarray results. Bisulfite sequencing (BS), COBRA analysis and real-time qPCR were used to confirm KSHV DNA methylation profiles at select loci. **A**: Three loci for which our MeDIP analysis had indicated profound methylation, and three loci which were predicted to be unmethylated were analyzed by bisulfite sequencing of BCBL1-derived DNA. The global BCBL1 MeDIP methylation profile and the location of sequenced regions are shown for reference at the top. The results of the bisulfite sequencing are shown underneath, where closed and open circles indicate methylated and unmethylated CpG motifs, respectively. The nucleotide positions indicate the position of the first and the last CpG motifs within the KSHV reference genome (NC_009333). **B–E**: Confirmation of DNA methylation profiles at the genomic ORF23 locus in PEL cells (HBL6, AP3 and BCBL1), long-term *in vitro* infected endothelial SLK cells (SLKp), SLK cultures 5 days after *de novo* infection (SLK-5dpi), *in vitro* methylated or unmethylated KSHV bacmids (BacM and Bac, respectively), and virion DNA. The methylation profiles of the samples investigated by MeDIP are shown in **B**. Black lines indicate the regions for which COBRA analysis and bisulfite sequencing of genomic DNA, or real-time qPCR of MeDIP samples were performed. **C**: Real-time qPCR was performed to quantify immunoprecipitated DNA from three independent MeDIP experiments. Values were calculated as percent of the input and were normalized to an internal control consisting of methylated plasmid DNA (pCR2.1) spiked into each sample prior to MeDIP. **D+E**: the region indicated in B was PCR-amplified from bisulfite converted DNA and subjected to a COBRA assay (**D**) or bisulfite sequencing (**E**). Cleavage of bisulfite converted DNA at the TaqI sites indicated by arrows requires methylation of the corresponding CpG motif. The CpG profiles as shown in E were determined by bulk sequencing reactions except for the samples labeled SLKp #1 and #2, which represent two individual clones from the SLKp line.

In order to confirm that the relative MeDIP values identified during our microarray-based analysis are indeed an accurate measure of CpG methylation levels, we investigated a number of loci using independent methods. First, based on our analysis of BCBL1 cells, we chose 3 loci which had registered as being strongly methylated, and another 3 for which our initial analysis had suggested the absence of DNA methylation. As shown in Figure 3A, bulk bisulfite sequencing established near-complete methylation at the former and absence of methylation at the latter loci. We selected one of the loci (labeled 1 in Figure 3A) which had shown differential methylation in PEL and SLKp cells for further analysis. Figure 3B shows an enlarged representation of the corresponding section of the KSHV genome, along with the original MeDIP array data. As shown in Figure 3C, quantitative real-time PCR amplification of an ∼100 bp segment at the center of the region (indicated by the black bar labeled “qPCR” in Figure 3B) confirmed the overall lower degree of methylation in SLKp cells and suggested an intermediate degree of methylation in AP3 cells, which is in accordance with the array data for this position. As discussed later, we also investigated *de novo* infected SLK cells at 5 days post infection (SLK-5dpi), which showed very little evidence of methylation. Next, we performed a PCR amplification of bisulfite converted total DNA from all samples and subjected the amplified region to digestion with the restriction enzyme TaqI (combined bisulfite restriction analysis assay, COBRA). The recognition sequence of TaqI contains a CpG motif, and as only methylated cytosine residues are preserved during bisulfite conversion, absence of DNA methylation at the restriction site leads to TaqI resistance. As shown in Figure 3D, unmethylated bacmid DNA as well as DNA isolated from KSHV virions were completely unmethylated and hence resistant to TaqI restriction. Likewise, DNA from freshly infected SLK cells remained intact. In contrast, the amplification products from the *in vitro* methylated bacmid as well as BCBL1 and HBL6 were completely cleaved, in agreement with methylation of all 4 TaqI sites, and the products from AP3 cells and SLKp cells were incompletely digested, indicating an intermediate level of methylation. The latter contained a significant amount of undigested product, suggesting that the material represents a mixture of methylated and unmethylated DNA, presumably due to clonal differences in the original single cell clones. We hence determined the specific sequence of bisulfite converted DNA from two individual clones, and subjected the remaining samples to bulk sequencing. The results of this analysis are shown in Figure 3E and are in perfect accord with our COBRA analysis and MeDIP array results. Indeed, the two investigated SLKp clones showed differential methylation patterns at this particular locus, thus explaining the observed restriction patterns. We hence conclude that our array analysis accurately reflects CpG DNA methylation within the KSHV genome.

**Figure 4 ppat-1000935-g004:**
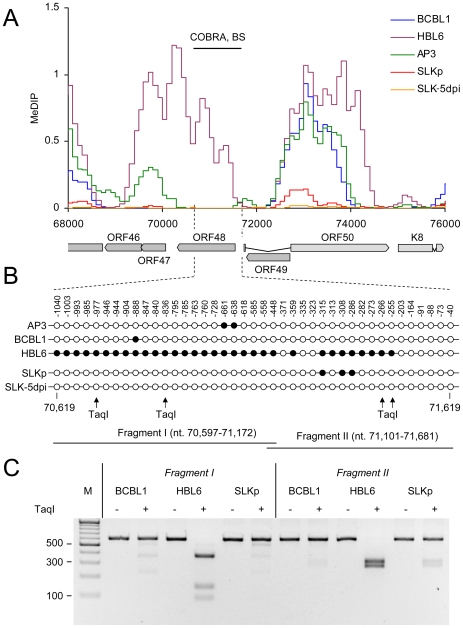
DNA Methylation at the ORF50 promoter. **A**: Methylation profiles of KSHV infected cells at the ORF50 promoter (see legend to Figure 3 for abbreviations). The region investigated by COBRA and bisulfite sequencing is indicated by the black bar above and hashed lines underneath the graph. **B**: Results of bisulfite sequencing of genomic DNA from BCBL1, HBL6 or SLKp cells. Closed and open circles indicate methylated and unmethylated CpG motifs, respectively. Numbers above each circle indicate the position of the motif relative to the ORF50 transcriptional start. The nucleotide positions shown underneath indicate the position of the first and the last CpG motifs within the KSHV reference genome (NC_009333). The position of TaqI restriction sites in bisulfite converted DNA is indicated by arrows (conservation of the sites requires methylation of the corresponding CpG motif). The black bars labeled “Fragment I” and “II” represent the two overlapping PCR fragments which were amplified and sequenced, and which were further analyzed by COBRA as shown in **C**.

As noted before, the global methylation patterns in PEL and SLKp cells were highly similar: If a locus was found to be methylated in one of the samples it tended to be methylated also in the others. Only very few loci were methylated in only one sample. Interestingly, the most prominent locus which showed differential methylation was a region encompassing nts. 70500 to 71700, which includes the promoter governing expression of ORF50/Rta. Our analysis suggested profound methylation in the HBL6 line, but very little or no methylation in AP3, BCBL1 and SLKp cells (see Figure 2 (second panel) and enlarged depiction of the ORF50 locus in Figure 4A). This was surprising, as the ORF50 promoter has been previously reported to be abundantly methylated in BCBL1 cells [26]. To confirm our results, we performed bulk bisulfite sequencing of the region extending from the transcriptional start of ORF50 to a position approximately 1100 bp upstream (nts. 70597 to 71681) with DNA isolated from AP3, BCBL1, HBL6, SLK-5dpi and SLKp and cells (Figure 4B). Additionally, we subjected the two overlapping amplification products from BCBL1, SLKp and HBL6 cultures to COBRA analysis (Figure 4C). The results clearly confirmed our MeDIP results and revealed near complete CpG methylation of the ORF50 promoter region in the HBL6 line, but no or only sporadic methylation in all other cells. We currently do not know the reason for the different BCBL1 methylation patterns detected in our study and that performed by Chen et al. [26]. It is possible that Chen and colleagues employed a different sub-clone of the BCBL1 line, or that the lines may have diverged while being cultured in the two labs. The methylation patterns we detected in HBL6 cells are very similar (but not identical) to those described by Chen et al., and thus an agreement between both studies is that such patterns can principally evolve in PEL cells. However, regardless of the reasons for the different findings, our results clearly show that methylation of the ORF50 promoter is not a principal requirement for the maintenance of latency in PEL cells or *in vitro* infected endothelial cells lines. As the majority of cells in PEL and KS tumors are latently infected with KSHV, our findings may also provide an alternative explanation for the observation that the ORF50 promoter was found to be not or only poorly methylated in the majority of clones derived from such tissues [26].

**Figure 5 ppat-1000935-g005:**
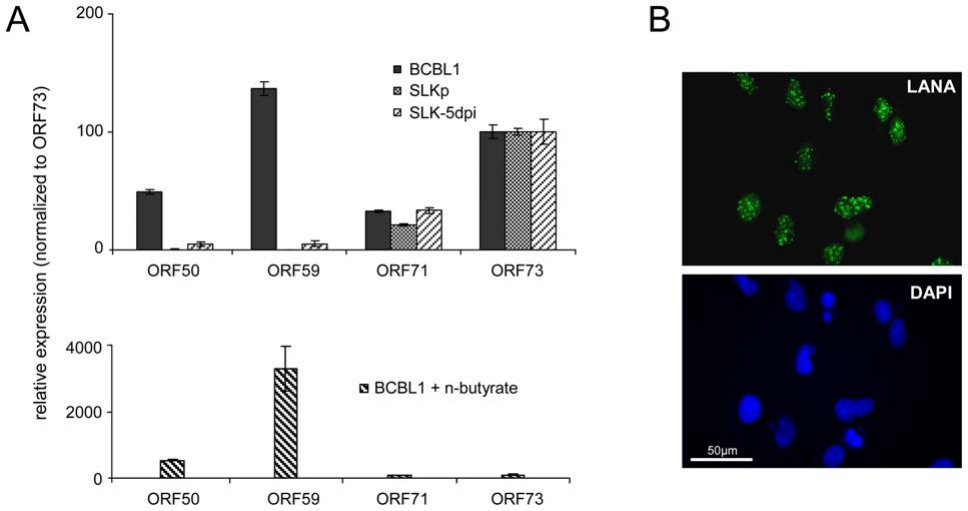
Latent KSHV expression patterns of SLK*_P_* and *de novo* infected SLK cells. **A**: Expression of select latent (ORF71, ORF73) and lytic (ORF50, ORF59) transcripts was analyzed by quantitative RT-PCR in BCBL1 cells, long-term infected SLK*_P_* cells and *de novo* infected SLK cultures at day 5 post infection (SLK-5dpi). Levels were normalized to represent expression relative to ORF73, which is expressed during the latent as well as the lytic cycle. Compared to BCBL1, SLK-5dpi cells show little expression of lytic antigens, and expression was undetectable in SLKp cells. The detection of lytic transcripts in latent BCBL1 cultures is due to the low percentage (less than 1%) of cells which undergo spontaneous lytic reactivation. The percentage of lytic cells and thus transcript levels can be increased by treatment such as sodium butyrate (lower panel). Spontaneously reactivating cells are completely absent from SLKp cells, which is in accordance with the lack of detectable lytic gene expression. **B**: Immunofluorescence staining of SLK-5dpi cultures for LANA, the product of ORF73. DAPI staining is shown in the lower panel. More than 90% of the cells tested positive for LANA, while expression of the lytic DNA polymerase processivity factor encoded by ORF59 could be detected in less than 0.01% of cells (compare also left column in Figure 9F).

SLKp cells exhibited global methylation patterns which were near-identical to those seen in the BCBL1 line, but were characterized by a significantly lower absolute level of DNA methylation (approximately 1/5th of that seen in BCBL1 cells). This observation suggested to us that DNA methylation of KSHV episomes may progress slowly over time; hence the lower overall extend of methylation would be a result of the comparatively short period of time (approx. 8 months, see above) that has elapsed since the SLKp cells were originally infected. We therefore analyzed SLK cultures which had been freshly infected with KSHV. We choose a time point of 5 days post-infection for our analysis; at this time point, the cultures have adopted latent expression patterns and sporadic lytic cells are found only at very low frequency (∼0.01%) [38], [39]. Quantitative RT-PCR (Figure 5A) and immunofluorescence analysis (Figure 5B) confirmed efficient infection and absence of lytic gene expression (note that the relatively high basal levels of lytic ORF50 and ORF59 transcripts in latent BCBL1 cultures (Figure 5A, top panel) can be significantly upregulated by lytic cycle induction (bottom panel); they stem from the small number (approximately 0.3%, see Figure S4) of spontaneously reactivating cells present in uninduced BCBL1 cultures). Indeed, our array-based MeDIP analysis of global DNA methylation patterns of SLK-5dpi cultures revealed very little DNA methylation at this early time point of infection (see graphs labeled SLK-5dpi in Figures 2, 3B and 4A), reaching, on average, less than 1% of the levels observed for the positive control. DNA methylation was also virtually absent from the ORF50 promoter, in spite of the fact that the infected cultures had established a strictly latent infection. These findings support our hypothesis that, although DNA methylation may reinforce latent gene expression patterns at late timepoints of infection, ORF50 promoter methylation is principally not required to abolish or prevent Rta expression during KSHV latency.

### Histone modification patterns of latent KSHV episomes

**Figure 6:**
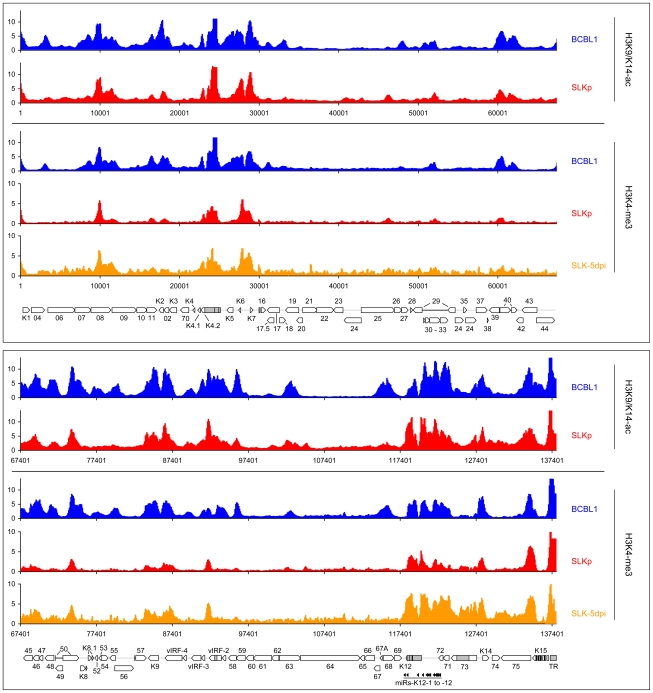
Global patterns of H3K9/K14 Acetylation and H3K4 tri-methylation on latent KSHV genomes. Global patterns of H3K9/K14 Acetylation (H3K9/K14-ac) of KSHV genomes in BCBL1 and SLKp cells, as well as H3K4 tri-methylation (H3K4-me3) patterns in BCBL1, SLKp and SLK cultures at 5 days post infection (SLK-5dpi) were analyzed by ChIP-on-chip assays as described in the text. Values shown on the y-axis represent relative enrichment of normalized signals from the immunoprecipitated material over input, calculated for overlapping sequence windows of 250 bp by averaging the values from all matching probes, as described in the legend to Figure 1 and the Material & Methods section. See legend to Figure 1 for explanation of map elements displayed at the bottom of each panel.

Given the absence of DNA methylation in our SLK-5dpi samples, we deemed it unlikely that this modification governs early KSHV latency expression patterns, and hypothesized that such patterns might rather be governed by histone modifications. Herpesvirus genomes are known to become rapidly chromatinized [40] after host cell entry, and the deposition of histone modifications is therefore expected to represent a much more dynamic process than DNA methylation. To investigate this hypothesis, we performed chromatin immunoprecipitation (ChIP) experiments from BCBL1, SLKp cells or *de novo* infected SLK cultures and analyzed the precipitated DNA on our tiled microarrays using standard ChIP-on-chip protocols (see Material & Methods for details). First, we investigated the distribution of two modifications which are commonly associated with active chromatin, using antibodies which are specific for Histone H3 acetylated at lysine 9 and/or 14 (H3K9/K14-ac), or H3 molecules which are tri-methylated at lysine K4 (H3K4-me3). As shown in Figure 6 (see also Figure S2 for a more detailed view), in BCBL1 as well as SLKp cells we observed global modification patterns which were highly similar when comparing any pairwise combination of either histone modification or cell line, with correlation coefficients ranging from 0.709 to 0.894 (Table 2). Furthermore, investigation of H3K4-me3 patterns in SLK-5dpi cultures showed that these patterns were indeed already fully established 5 days after *de novo* infection. Comparison with the previously observed CpG methylation patterns revealed a marked negative correlation between these histone modifications and DNA methylation, as most of the regions which had been found to be poorly methylated in BCBL1 or SLKp cells compared to the positive control showed abundant deposition of active histone marks. In accordance with the overall higher degree of DNA methylation, this negative correlation was most obvious in BCBL1 cells (Pearson correlation coefficient = −0.530, see Table 2), but could also be clearly observed in SLKp cells (correlation coefficient = −0.263). Interestingly, while the highest density of CpG motifs within the KSHV genome is found at the terminal repeats (TR, see rightmost region of the KSHV map), this is also the region which showed the highest levels of H3K9/K14-ac and H3K4-me3 enrichment. The latter is in agreement with the observation that, in spite of the high number of potential methylation sites, MeDIP signals were absent from this region (Figure 2). In fact, in bulk bisulfite sequencing reactions, we were unable to identify any DNA methylation within the terminal repeats in SLKp or BCBL1 cells (data not shown). Hence, our data indicate that local deposition of active histone marks early during KSHV infection prevents the acquisition of DNA methylation over the ensuing cell divisions, ultimately leading to the establishment of the global methylation patterns as shown in Figure 2.

**Table 2:** Pearson correlation coefficients of DNA methylation and histone modification patterns.

	*MeDIP*			*H3K9/K14-ac*		*H3K4-me3*			*H3K27-me3*			*H3K9-me3*	
	*BCBL1*	*SLKp*	*SLK-5dpi*	*BCBL1*	*SLKp*	*BCBL1*	*SLKp*	*SLK-5dpi*	*BCBL1*	*SLKp*	*SLK-5dpi*	*BCBL1*	*SLKp*
***H3K9/K14-ac***													
*BCBL1*	−0.539	−0.321	−0.292										
*SLKp*	−0.384	−0.246	−0.189	0.848									
***H3K4-me3***													
*BCBL1*	−0.530	−0.341	−0.262	0.894	0.801								
*SLKp*	−0.263	−0.180	−0.095	0.709	0.824	0.772							
*SLK-5dpi*	−0.291	−0.119	−0.079	0.660	0.822	0.676	0.794						
***H3K27-me3***													
*BCBL1*	0.107	0.016	−0.013	−0.459	−0.565	−0.450	−0.428	−0.528					
*SLKp*	0.458	0.210	0.141	−0.430	−0.508	−0.376	−0.406	−0.482	0.619				
*SLK-5dpi*	0.414	0.233	0.183	−0.501	−0.613	−0.449	−0.415	−0.477	0.593	0.812			
***H3K9-me3***													
*BCBL1*	0.663	0.413	0.303	−0.379	−0.278	−0.267	−0.131	−0.203	−0.093	0.412	0.331		
*SLKp*	0.406	0.397	0.230	−0.263	−0.206	−0.151	−0.069	−0.095	−0.055	0.218	0.215	0.615	
*SLK-5dpi*	0.081	0.107	0.197	−0.061	−0.030	0.024	0.123	0.269	−0.009	0.078	0.302	0.130	0.185

*Note: correlation coefficients were calculated according to Pearson from the data shown in* *Figures 2* *,* *6* *and* *7* *. All data points are given in Dataset S1.*

**Figure 7:**
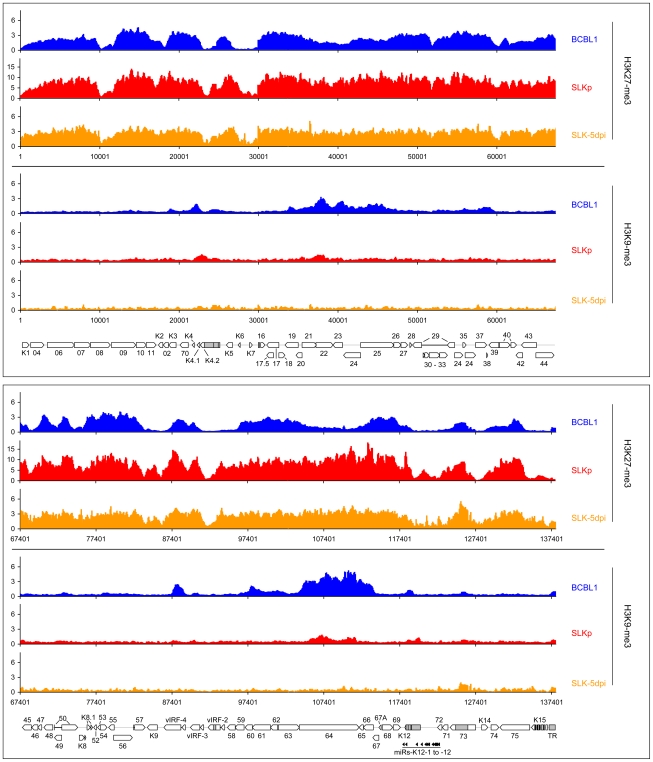
Global patterns of H3K27 and H3K9 tri-methylation on latent KSHV genomes. Global patterns of histone H3 tri-methylated at lysine 27 (H3K27-me3) or 9 (H3K9-me3) on KSHV genomes in BCBL1, SLKp cells as well as SLK cultures at 5 days post infection (SLK-5dpi) were analyzed by performing ChIP-on-chip assays as described in the text. Values shown on the y-axis represent relative enrichment of normalized signals from immunoprecipitated material over input, calculated for overlapping sequence windows of 250 bp by averaging the values from all matching probes, as described in the legend to Figure 1 and the Material & Methods section. See legend to Figure 1 for explanation of map elements displayed at the bottom of each panel.

While it may account for the evolution of the observed DNA methylation patterns, the distribution of H3K9/K14-ac and H3K4-me3 modifications provides no immediate explanation for the establishment of latent expression profiles, as these marks were present on many loci which are transcriptionally inactive during latency. Notably, this also includes the ORF50 promoter, a finding which is in accordance with the absence of DNA methylation at this location in BCBL1 as well as SLKp cells. We therefore reasoned that latency may be determined by the presence of repressive marks rather than the absence of activating ones. Therefore, we analyzed two modifications commonly associated with silent chromatin: Tri-methylation of lysine 9 of histone H3 (H3K9-me3), which is a hallmark of constitutive heterochromatin, and tri-methylation of lysine 27 (H3K27-me3), a modification which is typically seen in facultative heterochromatin. As shown in Figure 7 (lower graphs in each panel; see also Figure S3 for a more detailed view), in both BCBL1 and SLKp cells the H3K9-me3 modification was mainly restricted to two consecutive regions of the viral genome, spanning approximately nts. 33000 to 46000 (ORF19-ORF25) and 100400 to 114400 (ORF64- ORF67). Both of these regions had shown relative poor occupancy with acetylated histones in our previous assay (see Figure 6), which is in agreement with the fact that these modifications in general are mutually exclusive. While the H3K9-me3 modification was most prominently detected in BCBL1 cells, SLKp cells did display a markedly less distinct pattern, and the modification was barely detectable in SLK-5dpi cultures. The ORF50 promoter was devoid of trimethylated H3K9 in all samples. Hence, H3K9-me3 is unlikely to be a major regulator of latent gene expression, at least not in the early phase of infection when latency is first established. Our findings are in agreement with a previous study that had investigated a number of select loci in ChIP experiments, and had found little to no H3K9 methylation at any of them [41].

Next, we analyzed the distribution of the H3K27-me3 modification across the viral genome. H3K27 tri-methylation is carried out by EZH2, the enzymatic subunit of the polycomb PRC2 complex, leading to the recruitment of polycomb PRC1 complexes and thus gene silencing [29], [30], [31], [42], [43], [44]. Tri-methylation of H3K27 has been shown to play important roles in developmental and differentiation processes, cell cycle regulation, mammalian X chromosome inactivation, stem cell identity and cancer [31]. One characteristic of H3K27 methylation is that, in contrast to H3K9-me3, it can occupy promoters concurrently with activating modifications, specifically H3K4-me3 or H3K9-ac. Such regions are termed “bivalent” domains and have been found to be specifically enriched in embryonic stem cells, where they often occupy promoters which encode key factors involved in developmental regulation [42]. The presence of H3K27 methylation keeps these promoters silent in undifferentiated cells, but the chromatin remains in a “poised” state due to the simultaneous presence of activating marks. Decreasing levels of H3K27-me3 during the onset of differentiation allows such promoters to rapidly revert to an active state, hence further commiting the cell to terminal differentiation [42]. As shown at the top of each panel in Figure 7, our analysis revealed that latent KSHV episomes in the BCBL1 and SLKp lines as well as SLK-5dpi cells were subject to abundant H3K27 tri-methylation. The modification was detected virtually across the complete genome, although most regions which had been found enriched in H3K9/K14-ac and H3K4-me3 modifications tended to be tri-methylated at H3K27 to a lesser extend (compare with Figure 6, see also correlation coefficients in Table 1). A number of loci, however, displayed the hallmarks of bivalent chromatin, i.e. simultaneous presence H3K27-me3 and activating marks. Interestingly, the ORF50 promoter featured prominently among these regions, whereas the major latency promoter upstream of ORF73 showed very little or no H3K27 tri-methylation in all three samples. Importantly, in contrast to H3K9-me3, the H3K27-me3 patterns were already present 5 days after *de novo* infection of SLK cultures. This also includes the ORF50 promoter, and our data thus suggest that a poised state of repression is imposed upon the ORF50 promoter early during the establishment of latency. Interestingly, two studies have recently found this modification to be present on herpes simplex virus genomes during latent infection in dorsal root or trigeminal ganglia [45], [46]. Although only a small number of select promoters were investigated, this may indicate a general role for this modification during herpesvirus latency.

**Figure 8:**
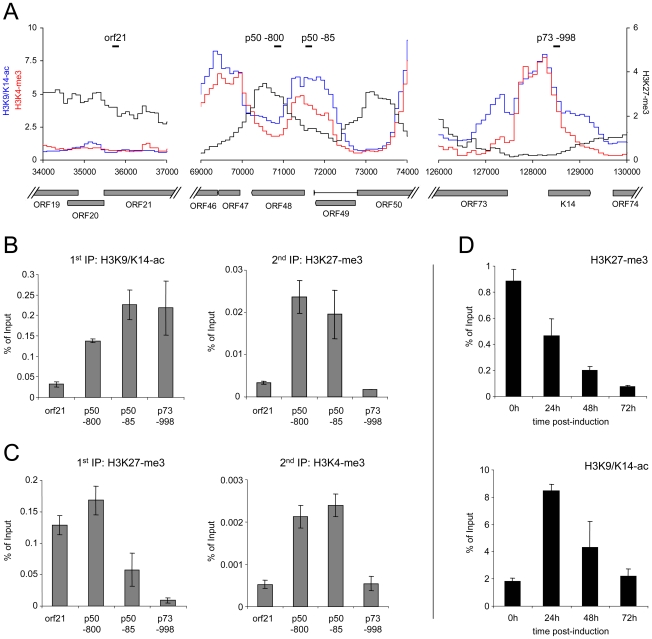
Bivalent histone modification patterns at the ORF50 promoter are reversed upon induction of the lytic cycle. **A**: Profiles of H3K9/K14-ac (blue), H3K4-me3 (red) and H3K27-me3 (black) histone modifications at the ORF21 (left), ORF50 (center) and ORF73 (right) loci of BCBL1 cells. Black bars indicate the location of regions amplified by quantitative PCR in the sequential ChIP and lytic reactivation experiments shown in B–D. **B, C**: Sequential ChIP experiments carried out with antibodies directed against H3K9/K14-ac and H3K27-me3 during the first and second rounds of immunoprecipitation, respectively (**B**), or with antibodies against H3K27-me3 during the first ChIP, followed by H3K4-me3 specific antibodies for the second immunoprecipitation (**C**). For the first as well as the second round of immunoprecipitation, numbers on the y-axis indicate the percentage of recovered material relative to the total starting material (i.e., the amount of DNA which was used as the input during the first ChIP). **D**: Reversal of H3K27-me3 marks at the ORF50 promoter upon lytic reactivation. BCBL1 cells were treated with 0.3mM sodium butyrate to induce the lytic cycle. ChIP experiments were performed at the indicated time points to monitor changes in H3K27-me3 and H3K9/K14-ac modification patterns, using quantitative PCR with primers specific for the p50 −800 region as shown in A.

As all of the KSHV-infected lines investigated here contain multiple copies of the viral episome, it may appear possible that the simultaneous detection of activating marks and the H3K27-me3 modification could be due to the existence of distinct, but separate episome populations. We think this is very unlikely, as we have observed the same patterns in BCBL1 cells and *in vitro* infected SLK cells. Thus, if distinct populations exist, they would have to be re-established in the exact same stoichiometry upon a *de novo* infection. However, to also directly investigate the presence of bivalent histone modifications, we have performed a sequential ChIP from BCBL1 cells, followed by qPCR amplification of two regions within the ORF50 promoter. As controls, we investigated the latent promoter upstream of ORF73 (which exclusively carries activating marks) and a region within the coding region of ORF21 (which is subject to the H3K27-me3 modification, but is devoid of H3K9/K14-ac and H3K4-me3 marks). The location of the amplified regions and their histone modification profiles are depicted in Figure 8A. As shown in Figure 8B, when the first round of immunoprecipitation was carried out with an antibody specific for H3K9/K14-ac, the sequential ChIP using a H3K27-me3-specific antibody recovered material only from the ORF50 promoter, but neither of the two control regions. To confirm these results in the reverse direction, we also performed the first round of immunoprecipitation using the H3K27-me3-specific antibody and used a H3K4-me3 antibody for the second immunoprecipitation to probe for the presence of activating marks (Figure 8C). Again, while the ORF21 region was recovered in the first round of ChIP, only the ORF50-specific sequences registered in both immunoprecipitation experiments, thus demonstrating bivalent modification of this promoter.

If H3K27-me3 contributes to the silencing of the ORF50 promoter, these marks should also diminish upon lytic cycle induction. We therefore monitored the levels of H3K27-me3 during reactivation from latency. Indeed, sodium butyrate treatment of BCBL1 cells resulted in a progressive loss of H3K27-me3 at the ORF50 promoter, with a reduction to approximately 50%, 20% and 5% of the original levels after 24, 48 and 72h of treatment, respectively (Figure 8D). While this observation suggested efficient removal of H3K27-me3, the magnitude of the effect at 48h and 72h post induction was surprising, given that the treatment only reactivates about 20% of all cells in the cultures (as judged by staining for the late gene product ORF59). A possible explanation for this observation is that the rapidly increasing numbers of replication products (which are epigenetically naive) exaggerate the effect at late time points, as they will lead to a relative increase in the percentage of unmodified episomes within the cultures. However, the 24h time point precedes the replication phase and accumulation of newly synthesized/packaged genomes thus cannot be responsible for the H3K27-me3 decrease observed early after induction. To further substantiate this assumption, we also monitored H3K9/K14-ac levels at the ORF50 promoter. We reasoned that, if the above is correct, prior to the onset of DNA replication we should first see an increase of the histone acetylation levels, followed by a decline as more and more replicated genomes accumulate. As shown in Figure 8D, this is precisely what we observed. Thus, upon lytic cycle induction, a reduction of H3K27-me3 and an increase of H3K9/K14-ac levels occur simultaneously at the ORF50 promoter and precede the DNA replication phase.

**Figure 9:**
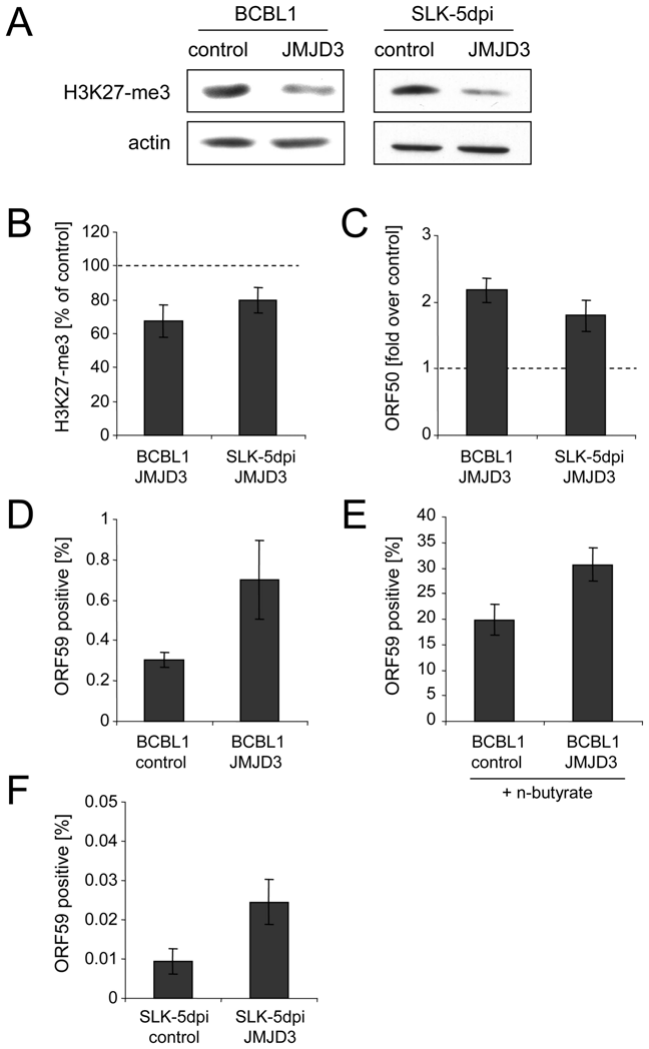
Consequences of JMJD3 expression in BCBL1 and *de novo* infected SLK cells. **A**: Reduction of global H3K27-me3 levels in BCBL1 (left) or SLK cells (right) after 2 weeks of transduction with a JMJD3-expressing retrovirus (right lanes in each panel) or with an empty control virus (left lanes). Western blots were simultaneously stained with antibodies specific for H3K27-me3 as well as actin. **B–F**: Analysis of JMJD3-transduced BCBL1 cells, as well as JMJD3-transduced SLK cells after 5 days of infection with KSHV. **B**: H3K27-me3 status of the ORF50 promoter, as judged by ChIP analysis followed by real-time qPCR with primers amplifying the p50 −800 region shown in Figure 8A. Values are shown as relative levels in JMJD3-transduced compared to the control cells, which were set to 100%. **C**: ORF50 transcription as judged by quantitative PCR. Values are given as fold transcript levels in JMJD3-transduced cells compared to control cultures (set to 1). **D** and **F**: Percentage of spontaneously reactivating cells (as judged by immunofluorescence staining for the product of ORF59) in JMJD3 expressing BCBL1 (D) or SLK (F) cells, or the corresponding control cultures. **E**: Percentage of ORF59 positive cells after induction of BCBL1 cells with sodium butyrate for 72h.

In order to investigate whether a reduction of H3K27-me3 also results in an increase of the number of lytically reactivated cells in the absence of chemical inducers, we next generated BCBL1 and SLK cells which were stably transduced with a retrovirus that expresses the H3K27-me3-specific demethylase JMJD3 [47]. After antibiotic selection of the cultures for 12 days, the SLK cells were additionally infected with KSHV and analyzed 5 days later. In both lines, while the ectopically expressed JMJD3 protein was barely detectable on western blots (data not shown), we nevertheless observed a reduction of total cellular H3K27-me3 levels of at least 50% (Figure 9A). The reduction was less pronounced on the ORF50 promoter, which still exhibited about 70% and 80% of the H3K27-me3 levels seen in the vector controls of BCBL1 and SLK-5dpi cells, respectively (Figure 9B). However, as shown in Figure 9C, despite of the moderate degree of this reduction, both cultures showed a marked increase in the overall levels of ORF50 transcription, which reached approximately twice the values as in the control cultures. When we stained the JMJD3-transduced BCBL1 cultures for the late gene product ORF59, (Figure 9D) we furthermore observed a twofold increase in the percentage of spontaneously reactivated cells in the JMJD3-transduced BCBL1 cells (∼0.6%, compared to 0.3% in the control cultures). In addition, the JMJD3-transduced cells were also more responsive to lytic cycle induction by sodium butyrate treatment, resulting in the reactivation of 30% of the cultures (compared to approximately 20% in the control cultures; Figure 9E).

In comparison to PEL lines, SLK cells as well as most other *de novo* infected adherent cell lines exhibit an extremely low percentage of spontaneously reactivated cells [38], [39]. Such cells do also not respond to chemical inducers which reactivate PEL cells (e.g. phorbol esters), although the lytic cycle can be induced by ectopic Rta/ORF50 overexpression [38]. Their low frequency notwithstanding, in addition to the elevated ORF50 transcript levels we also observed an approximately threefold increase of the number of spontaneously reactivated cells in JMJD3-transduced SLK-5dpi cultures (Figure 9E). Taken together, the above data suggest an important role for the H3K27-me3 histone modification during latent infection with KSHV.

While our analysis has revealed highly distinct patterns of DNA and histone modifications, the question remains which factors determine such patterns. The molecular requirements for the recruitment of PRC2/EZH2 methyltransferase complexes in mammals are poorly understood, and so far no simple sequence motifs recognized by these complexes have been described. The results from our early infected SLK cultures would seem to indicate that PRC2/EZH2 complexes are recruited to KSHV genomes in a more global fashion. However, it is also possible that the modification is initially established at a small number of loci and rapidly spreads to neighboring regions during the earliest stages of a latent infection. Whatever the mode of deposition, loci with lower levels of H3K27-me3 are ultimately confined to those regions which carry activating H3K9/K14-ac and H3K4-me3 marks which, clearly, are present not only on latency promoters. Two independent studies have noted the presence of such marks in latent cultures before, using ChIP in conjunction with either PCR for a number of select loci, or a global promoter microarray [24], [41]. Overall, the data from our high-resolution tiling arrays are in very good agreement with the findings reported in both studies. However, while Ellison and colleagues hypothesized that the detection of these marks may have been due to a low percentage of cells which undergo spontaneous reactivation, our study clearly shows that they are a hallmark of latent episomes: First and foremost, the patterns were not only detected in BCBL1 cells, but also in SLKp cultures, which are strictly latent and do not harbor any spontaneously reactivated cells. Second, for a low percentage of reactivated cells to leave a prominent footprint in the histone modification profile of the total population, one has to assume that they contain a disproportional high number of episomes which carry the lytic marks. Given the high copy number of *de novo* replicated genomes in reactivated cells, this seems a reasonable assumption (provided that the lytically replicated genomes inherit the parental modification patterns, and that histones are removed only immediately before packaging). However, this does not apply to DNA methylation (which is absent from replicated virion DNA). The fact that the global CpG methylation patterns observed in our study show a marked negative correlation with the activating histone marks thus strongly argues for their presence on latent episomes.

So what signals trigger the initial recruitment of activating marks at the earliest timepoints of infection? While this is, ultimately, an issue which will have to be resolved in future studies, when comparing our data with those of two recent studies which have performed genome-wide screens for Rta binding sites [24], [48], we noticed that a surprising number of sites mapped within or very close to the loci which were found to be enriched for activating histone marks during our investigation (see Figure S5). Interestingly, Rta is known to be expressed for a brief period of time within the first few hours of a *de novo* infection, before latency ensues [49]. Therefore, one attractive hypothesis is that binding of Rta may trigger the initial modification of H3K9/K14 and H3K4 at these sites. The onset of widespread H3K27 methylation then may lead to the silencing of the ORF50 promoter, establishing a poised state of repression which can be easily reverted upon reactivation. However, while this model may explain the initial deposition of activating marks, it does not provide a satisfactory explanation for their maintenance during the later stages of viral latency. So far, there is very little evidence for the propagation of activating histone marks through cell divisions; rather, it is thought that their preservation requires continuous transcriptional initiation. In contrast to DNA methylation or polycomb-associated H3K27-me3 marks, H3K9/K14-ac and H3K4-me3 modifications are therefore not considered inheritable (and, therefore in a strict sense also do not represent epigenetic modifications). Thus, even if Rta is indeed responsible for the initial establishment of H3K27-me3 and H3K9/K14-ac marks, due to its rapid eradication upon establishment of latent expression patterns it cannot be responsible for their long-term maintenance. One possible explanation is that these loci represent preferred binding sites not only for Rta, but also for constitutively expressed host transcription factors. In this scenario, host factors could sustain the poised state of repression at H3K27-me3 enriched promoters, but additional stimuli would be required to return them to an active state. There are, however, also a few loci which are rich in H3K9/K14-ac and H3K4-me3 marks, but display very little H3K27 tri-methylation or DNA methylation. These include not only the constitutively active latency promoter upstream of ORF73 as well as the locus encoding the KSHV miRNA-cluster, but also ORFs K5/K6/K7/nut-1, the region upstream of three of the four vIRFs (vIRF1/K9, vIRF-3 and vIRF-4) and the complete K15 gene region at the right end of the viral genome. vIRF-3 (also termed latency-associated antigen 2, LANA2) is known to be expressed in latent PEL cells [50] and K15 (which encodes the latency associated membrane protein/LAMP) has been originally identified as a latently expressed gene, although its expression is significantly upregulated during the lytic cycle [51]. Since the region immediately upstream of the K1 gene at the left side of the KSHV genome as well as the terminal repeats (shown only to the right of the map in Figures 2, 5 and 6; as the episome is circular they however also flank the left terminus of the genome) are also highly enriched in activating marks but display very little H3K27-me3, our data also support a previous report of K1 expression in latently infected cells [52]. However, as K1 transcription is strongly upregulated in lytic cells and LANA has been found to repress K1 gene expression [53], whether K1 is indeed expressed at significant levels during latency is currently unclear. Interestingly, the region upstream of the K2 gene also displayed a high ratio of activating vs. repressive marks in BCBL1 cells (which is less pronounced in SLKp or SLK-5dpi cells). K2 encodes v-IL6, a viral homologue of IL6 which supports B cell growth and blocks interferon responses [54], [55]. While v-IL6 has been reported to be expressed in latently infected PEL cells [54], [56], similar to K1 its expression is strongly upregulated by lytic cycle induction and it thus remains controversial whether it represents a latent gene.

Although latent transcripts of unknown promoter origin have also been identified in the broader region encompassing ORFs K4 to K7 [57], most of the remaining genes so far have not emerged as being latently expressed in experimental systems [58]. If continuous transcriptional initiation is required to maintain H3K9/K14-ac and H3K4-me3 marks, then additional factors may exist which stall RNA polymerase II at these loci. Alternatively, it is possible that above genes are transcribed during latency only at low level, or that their mRNAs are rapidly turned over such that they do not accumulate. Transactivation by Rta as well as transcript stabilization by other lytic gene products (e.g. the product of ORF57 ) may then allow efficient expression of these genes once the viral genome is committed to productive replication. Taken together, our data thus provide a rationale for the observation that some of the above genes have been found to be expressed at low level in latent cultures. Importantly, they may also help to understand how the host environment may modulate the latent gene expression program. For example, IFN-α treatment of PEL cells has been shown to result in the transactivation of the K2 promoter via IFN-stimulated response element (ISRE) sequences [55]. It has been suggested that this enables the virus to sense innate immune responses and modify its gene expression in order to block them, a model which is strongly supported by the observation that the K2 promoter appears to be already primed for expression in latently infected PEL cells.

Finally, there is also the question of the role of the profound DNA methylation which occurs at later stages of viral latency. It appears likely that these patterns are established as a consequence of the continuous presence of EZH2/PRC2 repressor complexes on viral DNA, as such complexes have been shown to directly recruit DNA methyltransferases (DNMTs) [59]. The absence of DNA methylation at loci which are devoid of H3K27-me3 would support this conclusion. Another contributing factor could be the delayed appearance of constitutive heterochromatin marks: While H3K9-me3 is restricted to a few regions, it may nevertheless support the recruitment of DNMTs to the viral genome (this may be especially the case at the ORF64 locus, which displays the highest levels of H3K9-me3 but only moderate levels of H3K27-me3). What are the functional consequences of DNA methylation? Currently, this is a question that is difficult to answer. Based on the fact that SLK cells establish latent expression patterns in the absence of DNA methylation, and that BCBL1 and AP3 cells maintain latency in spite of the lack of DNA methylation at the ORF50 promoter, one may be tempted to think that this epigenetic mark is of no fundamental importance during KSHV latency. However, this is a conclusion which cannot be drawn. Compared with SLKp cells, *de novo* infected SLK-5dpi cells indeed display elevated levels of lytic gene expression and a higher number of spontaneously reactivated cells (Figs. 5 and 9). However, the generally low transcript levels together with the scarcity of lytic cells even in SLK-5dpi cultures complicate any general conclusion. Likewise, as the cells do not respond to chemical treatment with reagents that induce the lytic cycle in PEL cells, comparative studies of reactivation are difficult to perform. Studies employing Rta overexpression (which can reactivate such cells [38]) may be feasible, but they would be of limited value as the ectopic expression would artificially override one of the most critical steps of lytic reactivation. With regard to PEL cells, more lines will have to be studied to conclude whether absence or presence of methylation marks at the ORF50 promoter has an impact on the percentage of spontaneously reactivated cells and/or the response of such lines to chemical agents which induce the lytic cycle. At present, although such differences certainly exist between many PEL lines, this could in large part or entirely be a consequence of host cell differences. Lastly, even if DNA methylation should turn out to have no significant additive effect over the presence of repressive histone marks *in vitro*, this may be fundamentally different *in vivo*. Although the physiological triggers which reactivate *in vivo* latency reservoirs (e.g. memory B cells) are poorly understood, they are very likely to be much more specific than the broad pleiotropic effects induced by chemical agents such as phorbol esters or sodium butyrate. It is thus very conceivable that DNA methylation may represent an additional, functionally important block which augments repressive histone marks and re-inforces latent expression patterns during long-term latency *in vivo*, but which may be of lesser consequence in *in vitro* models of viral infection.

Considering all of the above, many questions remain to be answered before the molecular mechanisms which govern establishment and maintenance of KSHV latency are fully understood. However, especially given the unexpected spatial and temporal patterns of histone modifications and DNA methylation revealed by our study, the data presented here provide important clues as to the host and viral factors which might be at work, and should greatly help to design further studies aimed at elucidating the role of epigenetic modifications during this crucial phase of the viral lifecycle.

## Materials and Methods

### Cell culture and *de novo* KSHV infection

The establishment of SLKp cells has been described before [39]. Briefly, endothelial SLK cells [60] were infected with KSHV *in vitro* and passaged for several weeks. Seven KSHV-positive single cell clones were selected from the long-term infected cultures and pooled to form the SLKp line. SLKp cells and the parental SLK line were cultured in DMEM supplemented with 10% fetal calf serum and penicillin-streptomycin (5 µg/ml). The KSHV-positive PEL cell lines BCBL1 [6], HBL6 [61] and AP3 [62] were cultured in RPMI 1640 medium (Invitrogen) supplemented with 10% fetal calf serum and penicillin-streptomycin at a final concentration of 5 µg/ml. Concentrated supernatants of infectious KSHV virions were harvested from lytically induced BCBL1 cells as described [39]. *De novo* infection of SLK cells was performed by incubating 2×10^5^ cells at 70% confluency for 2 hrs with 500 µl virus supernatant at a concentration of 1×10^8^ KSHV genome equivalents per ml (as determined by quantitative PCR) in the presence of 8 µg/ml polybrene in EGM-2 medium (Lonza). Generally, more than 95% of cells were infected, as judged by immunofluorescence analysis for LANA 48h after infection. For lytic reactivation of BCBL1 cells, sodium butyrate was added to the culture medium at a final concentration of 0.3 mM.

### Immunofluorescence and western blot analysis

Cells were fixated with 4% paraformaldehyde in PBS for 15 min, permeabilized with 2% Triton X-100 in PBS for 10 min, blocked with 3% BSA in PBS and incubated with primary antibodies specific for LANA or ORF59 (Advanced Biotechnologies: #13-211-100) in blocking solution for 2 hrs. Cells were washed three times with PBS and incubated with secondary antibodies (Alexa Fluor-555 goat anti mouse and −488 goat anti rabbit) for another 2 hrs and analyzed by fluorescence microscopy. Western blot analysis of total cell lysates was carried out by standard SDS-PAGE and immunoblot protocols, using antibodies directed against histone tri-methylated at lysine 27 (Upstate: #07-449) or, as a loading control, actin (Santa Cruz: #SC-8432).

### Retroviral expression of the H3K27 specific demethylase JMJD3

A retroviral JMJD3 expression construct was kindly provided by Paul Khavari [47]. The retroviral backbone MSCV (Clontech) was used as a negative control. Supernatants containing infectious viral particles were harvested 48 hrs post transfection of PhoenixGP cells (Nolan Laboratory, http://www.stanford.edu/group/nolan/). BCBL1 and SLK cells were transduced with recombinant retroviruses by spin inoculation at 300×g for 1 h, using undiluted supernatants in the presence of 8 µg/ml polybrene. After inoculation, cultures were maintained in medium containing 2 µg/ml puromycin for 12 days to select for transduced cells.

### Analysis of CpG methylation by bisulfite sequencing and COBRA

Bisulfite sequencing was performed using the EpiTect Bisulfite Kit (Qiagen), following the manufacturer's instructions. The method relies on a chemical reaction that leads to the conversion of all unmethylated cytosine residues to thymidines, allowing the identification of originally methylated cytosines after PCR amplification and sequencing of the locus of interest. The sequences of all bisulfite sequencing primers employed in this study are given in Table S1. PCR products were sequenced directly (bulk sequencing) using either the forward or reverse primer from the original amplification. CpG methylation patterns were extracted from the bulk sequencing data using the BiQ Analyzer v2.0 software [63]. A combined bisulfite restriction analysis, short COBRA assay, has been described before [64]. Briefly, PCR products from bisulfite treated samples were digested with the restriction enzyme TaqI (Fermentas) and resolved on an agarose gel (3%). TaqI recognizes the nucleotide sequence TCGA, which contains a CpG dinucleotide. After bisulfite conversion, the site is only preserved if the original CpG motif was methylated (note that the bisulfite conversion creates additional TaqI sites at methylated CpG motifs which are flanked by C and A residues, as the C in position −1 is converted to a T by the bisulfite reaction).

### Isolation, reverse transcription and PCR quantitation of RNA

RNA was isolated using the RNA-Bee (Tel-Test, Inc.) reagent. Contaminating DNA was removed by incubation with amplification grade DNase I (Invitrogen) and cDNA was prepared from random-primed RNA using Superscript III (Invitrogen) as per the manufacturer's instructions. Real-time quantitative PCR (qPCR) of cDNA or genomic DNA samples was performed using SensiMix SYBR Kit (Quantace) on a Rotorgene 6000 light cycler (Corbett Life Science). For quantitation, standard curves were created using dilutions of genomic BCBL1 DNA over a range of at least 10000×. The sequences of all primer pairs used in this study are given in Table S1.

### Chromatin Immunoprecipitation assay (ChIP)

ChIP analysis was performed as described by Si et al. [65] and recommended by the array manufacturer (Agilent Mammalian ChIP-on-chip protocol V10.0, May 2008), with some modifications. A detailed protocol of the procedure is given in Protocol S1. Briefly, chromatin from 5×10^6^ to 2×10^7^ cells was cross-linked with 1% formaldehyde. After quenching of the reaction by the addition of glycine, cells were lysed and nuclei were isolated by centrifugation. Chromatin was extracted from the isolated nuclei and fragmented by sonication using a Bioruptor (Diagenode) to an average length of 100–500 bp. A portion of the total chromatin sample was set aside for the later preparation of input controls. Material from 1×10^6^ cells was pre-cleared with salmon-sperm DNA protein-A agarose beads (Upstate) to reduce non-specific background and subjected to immunoprecipitation using 2 to 10 µg of antibodies specific for the histone modifications H3K9/K14-Ac (Upstate: #06-599), H3K4-me3 (Upstate: #04-745), H3K9-me3 (Upstate: #17-625) or H3K27-me3 (Upstate: #07-449). After incubation for 16 hrs, chromatin-immunocomplexes were precipitated by the addition of protein-A agarose, washed, eluted and de-crosslinked overnight at 65°C. DNA was purified by phenol-chloroform extraction and ethanol precipitation.

For preparation of input controls, 1/4^th^ of the amount of chromatin used in the immunoprecipitation reactions was employed. Input samples were treated in an identical manner as the immunoprecipitated samples, starting with the de-crosslinking step. Both samples were subsequently subjected to whole genome amplification and labeling using a linker mediated PCR protocol (Agilent Mammalian ChIP-on-chip protocol V10.0, May 2008), followed by microarray hybridization.

### Sequential ChIP assay

Colocalization of bivalent histone marks was measured by use of a sequential ChIP assay. Prior to the first IP antibodies were incubated with protein-A agarose beads (Upstate) for 2 hrs at 4°C. Antibody bead complexes were washed twice with 0.2 M triethanolamine buffer (Sigma). Beads and antibodies were coupled covalently by incubation with 20 mM dimethyl pimelimidate dihydrochloride (DMP, Sigma) in 0.2 M triethanolamine buffer on a rotating wheel for 30 min at RT, and the reaction was stopped by washing with 50 mM Tris-HCl (pH 7.5). Uncoupled antibodies were removed by pre-elution with 0.1 M acetic acid (pH 3.0) for 5 min at RT. Beads were incubated with diluted chromatin samples for 16 hrs at 4°C. Washing and elution was performed in a identical manner as described above for the standard ChIP assay. 1/16th of the precipitated chromatin was de-crosslinked and was used to determine the efficiency of the first IP by real-time qPCR. The remainder was employed as the input for the second IP, which again was performed according to the standard ChIP protocol. Results were calculated as percent of the original input, i.e. the total amount of DNA which was subjected to the first round of immunoprecipitation.

### Methylated DNA Immunoprecipitation assay (MeDIP)

MeDIP analysis was essentially performed as described before [35], [66], [67], [68], with some modifications. A detailed protocol is given in Protocol S2. Briefly, highly pure genomic DNA served as an input in the MeDIP procedure. Negative and positive controls were prepared by mixing genomic DNA from KSHV-negative SLK cells with unmethylated or *in vitro* methylated KSHV bacmid DNA [36], respectively. The ratio of viral vs. cellular DNA was selected such that it mimics the episome content typically seen in KSHV-infected PEL cell lines and the SLKp line (approx. 30–40 copies per cell). All DNA samples were sonicated to an average fragment size of 100–500 bp using a Bioruptor (Diagenode). In order to allow quantification and normalization of the data, a constant amount (0.2 ng) of *in vitro* methylated pCR2.1 plasmid was added per 5 µg of the sheared DNA. 1 µg of the sample was set aside as an input control, and the remainder was subjected to immunoprecipitation using 2.5 µg of a 5′-methylcytidine specific antibody (MAb-5MECYT-100, Diagenode). The precipitated immunocomplexes were harvested using Dynabeads M-280 Sheep anti-Mouse IgG (Invitrogen). After washing, DNA was eluted and purified by phenol-chloroform extraction and ethanol precipitation. Input control samples were treated identical to the IP samples, starting with the ethanol precipitation step. The samples were subsequently analyzed by qPCR and/or microarray hybridization.

### *In vitro* methylation of DNA

For control and normalization purposes, we prepared *in vitro* methylated DNA from a bacmid containing the complete KSHV genome [36] or the pCR2.1 vector (Invitrogen). DNA was methylated by incubating 15 µg of DNA with 40 units of the CpG methyltransferase M.SssI (NEB) for 2 hrs in 1× NEBuffer 2 containing 160 µM S-adenosylmethionine (SAM). Fresh SAM was added and reactions were incubated for another 2 hrs. DNA was purified and the reaction was repeated once to ensure complete methylation. Complete methylation was confirmed by restriction analysis using methylation sensitive enzymes (HpaII and MspI, Fermentas), and/or bisulfite sequencing of specific loci.

### Microarray design

Custom high-resolution KSHV microarrays were designed by shifting a sequence window of 60 nt. across both strands of the prototypic KSHV sequence (type P, accession number NC_009333) as well as the terminal repeat unit (KSU86666). Probes with a length between 45 and 60 nucleotides were selected from these windows such that their melting temperature was close to the optimal Tm of 80°C. To also ensure complete coverage of type M KSHV strains, the resulting probe sets were aligned to the type M reference sequence (NC_003409) and additional probes were designed in a identical manner for all regions with a length of 80 or more nucleotides which were not already covered by the original probe set. The length of all probes was subsequently adjusted to 60 nucleotides using sequences from a common linker (ATAACCGACGCCTAA), and each probe was synthesized in duplicate on Agilent 8×15k custom microarrays. For normalization purposes, the array also contains probe sets which were generated in an identical manner to cover the adenovirus type 5 genome (AY339865) as well as the pCR2.1 plasmid (Invitrogen).

### Microarray sample labeling and hybridization

500 ng of MeDIP-input or ChIP-input controls, 500 ng of immunoprecipitated ChIP material and all of the MeDIP material were labeled with Cy3 and Cy5 using Agilent Genomic DNA Labeling Kit PLUS according to Agilent's recommendations. For normalization purposes, 0.1 ng of Adenovirus Type 5 DNA were added to each samples prior to the labeling procedure. After labeling, samples were purified using Microcon YM-30 filter columns (Milipore), blocked using Agilent blocking solution and human cot-1 DNA (Invitrogen), and hybridized using the Agilent Oligo aCGH/ChIP-on-chip Hybridization Kit at 65°C for 24 hrs in a rotating oven. Arrays were washed once with Oligo aCGH/ChIP-on-chip Wash Buffer 1 (Agilent 5188–5221) at RT for 5 min and in Oligo aCGH/ChIP-on-chip Wash Buffer 2 (Agilent 5188–5222) at 37°C for 1 min and scanned using a GenePix Personal 4100A scanner (Axon Instruments).

### Microarray data analysis and normalization

Primary array analysis and data normalization was carried out using GenePix Pro 6.0 software (Axon Instruments). All MeDIP datasets were normalized using the methylated pCR2.1 plasmid which had been added to the samples prior to the immunoprecipitation, thus controlling for differences in MeDIP efficiency as well as labeling and array hybridization. Both channels were adjusted such that the average ratio of input vs. MeDIP signals across all pCR2.1-specific spots was 1. Similarly, ChIP datasets were normalized using the adenovirus type 5 DNA that was added as a spike-in prior to labeling, hence correcting for errors during labeling, hybridization or scanning of the samples. To eliminate false positive spots, we hybridized DNA from KSHV-negative SLK cells and identified all probes which exhibited high levels of background hybridization (i.e., fluorescence levels that exceeded the mean value plus 1× the standard deviation of all KSHV-specific spots on the negative control array). These probes (which mapped almost exclusively to repeat regions) were permanently flagged in all datasets and not used for further analysis. While our arrays carry probes specific for the M and P types of the KSHV genome, the KSHV genomes from the BCBL1 and AP3 lines have not been fully sequenced and thus may deviate from the reference genomes at a few locations. To control for such sequence differences, we flagged all spots which exhibited fluorescence levels which did not exceed a background fluorescence threshold in the input channel, which was set to the mean fluorescence plus twice the standard deviation of all negative control features (i.e. empty array features as well as spots containing irrelevant sequences, corresponding to all Agilent probes in the datasets which are labeled with “NC2_” and “(-)3xSLv1”). Note that, if sequence diversification leads to only a reduction of hybridization efficiency (e.g. due to single nucleotide polymorphism, which will not abolish hybridization), this will not falsify our results as the hybridization efficiency will be reduced in input as well as the immunoprecipitated sample; the ratio will thus be unaffected.

In addition to above quality controls, in each dataset we flagged all probes which exhibited more than 30% variance between duplicate spots. The 30% threshold corresponds to the mean variance plus twice the standard deviation exhibited by all KSHV-specific probes in all MeDIP experiments, thus removing all probes which show a significantly increased variance between individual spot repeats. MeDIP data were furthermore corrected by subtracting from each probe-specific signal the value observed in the negative control, i.e. the MeDIP sample representing the unmethylated KSHV bacmid in the background of cellular DNA. After normalization, an enrichment score was calculated for each of the probes, represented by the ratio of fluorescence signal intensities in the immunoprecipitated samples relative to the input control. As the average length of the immunoprecipitated MeDIP and ChIP fragments (100 to 500 bp) is greater than that of the tiled probes (45 to 60 nucleotides), the resolution of our analysis was limited by the fragment length rather than the array design. To account for this fact, the data presented in Figures 2 to [Fig ppat-1000935-g003] [Fig ppat-1000935-g004] [Fig ppat-1000935-g005] 6, 7 and 8 were calculated by tiling overlapping sequence windows of 250 nucleotides across the KSHV genome, using a step size of 100 nucleotides to advance each window. The type M reference sequence (NC_003409) was used for the HBL6 line, whereas the type P genome (NC_009333) was used for all other cells. The KSHV specific probes were subsequently blasted against the window sequences, and each window was awarded an enrichment score represented by the average score of all probes which showed more than 90% identity with either strand of its sequence. All scores (which were also used to calculate the Pearson correlation coefficients presented in Tables 1 and 2) are given in the Dataset S1. All raw data, including the original GPR files as well as sequence and match location(s) of individual probes are available from the Gene Expression Omnibus (GEO) Database at http://www.ncbi.nlm.nih.gov/geo, under accession number GSE19907.

## Supporting Information

Table S1 KSHV-specific PCR primers used in this study.
(0.08 MB DOC)

Protocol S1 ChIP Protocol.
(0.03 MB DOC)

Protocol S2 MeDIP Protocol.
(0.04 MB DOC)

Dataset S1 Dataset containing all datapoints used for Figures 2 to [Fig ppat-1000935-g003] [Fig ppat-1000935-g004] [Fig ppat-1000935-g005] 6, 7 and 8, and for the calculation of correlation coefficients given in Tables 1 and 2.
(0.60 MB XLS)

Figure S1 Global DNA methylation patterns of latent KSHV genomes (Higher magnification of data presented in Figure 2).
(0.50 MB PDF)

Figure S2 Global patterns of H3K9/K14 Acetylation and H3K4 tri-methylation on latent KSHV genomes (Higher magnification of data presented in Figure 6).
(0.41 MB PDF)

Figure S3 Global patterns of H3K27 and H3K9 tri-methylation on latent KSHV genomes (Higher magnification of data presented in Figure 7).
(0.44 MB PDF)

Figure S4 Spontaneous reactivation in BCBL1 cells. BCBL1 cells were analyzed by immunofluorescence for the expression of the ORF59 gene product. A phase contrast image (PC) is shown to the left, and an enlarged overlay of the section framed by the white rectangle is shown at the bottom.
(3.17 MB TIF)

Figure S5 Rta binding sites and global patterns of H3K4-me3 on latent KSHV genomes. H3K4 tri-methylation (H3K4-me3) patterns in BCBL1, SLKp cells as well as SLK cultures at 5 days post infection (SLK-5dpi) are shown as described in the legend to Figure 6. The location of regions which were found to harbor Rta binding sites in genome-wide screens performed by Chen et al. [48] or Ellison et al. [24] is indicated by dotted lines. The labeling above the lines indicates whether these regions were identified by Chen et al. (labelled “1”), Ellison et al. (“2”), or in both studies (“1,2”).
(1.02 MB TIF)
