# Supplementary material for: The Epigenetic Landscape of Latent Kaposi Sarcoma-Associated Herpesvirus Genomes
Source: PLoS Pathog. 2010 Jun 3;6(6):e1000935. doi: 10.1371/journal.ppat.1000935 (PMC2880564; doi:10.1371/journal.ppat.1000935)
Supplement: Table S1 — KSHV-specific PCR primers used in this study. (0.08 MB DOC) [file ppat.1000935.s001.doc]

**Table S1**

*KSHV-specific primers used in this study.*

| Application^a^ |  | Primer name |  | Sequence |  | Fig.3^b^ |
| --- | --- | --- | --- | --- | --- | --- |
| MeDIP- / RT-qPCR |  | ORF23fw |  | ACACGACACGATGTTTTCCA |  |  |
| MeDIP- / RT-qPCR |  | ORF23rv |  | TCATGGAGCGTGCTAACAAC |  |  |
| RT-qPCR |  | ORF59fw |  | GAACCTTTTGCGAAGACTCG |  |  |
| RT-qPCR |  | ORF59rv |  | TGCCAATCAGGTGACGTAAA |  |  |
| RT-qPCR |  | K1fw |  | CGGTTTGCTTTCGAGGACTA |  |  |
| RT-qPCR |  | K1rv |  | ATACCAGGATGTTGGCAAGG |  |  |
| RT-qPCR |  | ORF71fw |  | GGCGATAGTGTTGGGAGTGT |  |  |
| RT-qPCR |  | ORF71rv |  | GGATGCCCTAATGTCAATGC |  |  |
| MeDIP- / RT-qPCR |  | ORF73fw |  | TGGGTGAGTGTGGAGGTGTA |  |  |
| MeDIP- / RT-qPCR |  | ORF73rv |  | CCACCGCTTTCAAGTCCTAC |  |  |
| BS / COBRA |  | ORF23CTfw |  | TTATAAGTATTTTGGAATAATTTGGG |  | 1 |
| BS / COBRA |  | ORF23CTrv |  | TCAAACCAAATCTATACTAAAACACA | | 1 |
| BS |  | ORF43CTfw |  | TTAGTTGTAGAGAGGGGTTTTGTAA |  | 2 |
| BS |  | ORF43CTrv |  | CAAAACTTTACCAACTCCTAAACAC |  | 2 |
| BS |  | ORF50inCTfw |  | AAATAGATGGTTGAATAGGTGATT |  | 3 |
| BS |  | ORF50inCTrv |  | AAACATTTAACCTTCATTTCAATA |  | 3 |
| BS |  | ORFK8CTfw |  | GATGAAGTTGTTATTGAGGAAGATT |  | 4 |
| BS |  | ORFK8CTrv |  | ACAAAAACAAAAAAAAACAAAACAT |  | 4 |
| BS |  | miRNA1CTfw |  | ATATATGGGATTTTGGGTAGGATAG |  | 5 |
| BS |  | miRNA1CTrv |  | AATAACCTTAAAAATCCTACCTCCA |  | 5 |
| BS |  | Pro73CTfw |  | TTTTTGATTGGTGTTTTAGGTAG |  | 6 |
| BS |  | Pro73CTrv |  | AAAAATACACAAATAACAACCCTC |  | 6 |
| BS / COBRA |  | ORF50CT4fw |  | GTGTTTTATTATTTTTATAG |  |  |
| BS / COBRA |  | ORF50CT4rv |  | CATCTAACATAACTTTAATC |  |  |
| BS / COBRA |  | ORF50CT5fw |  | GTGGGTGATTTTTTTTATTA |  |  |
| BS / COBRA |  | ORF50CT5rv |  | TAAACAATATTCTCACAACA |  |  |
| ChIP-qPCR |  | ORF21fw |  | AATGCACGACAACTCCCTCT |  |  |
| ChIP-qPCR |  | ORF21rv |  | GACAACCGACTGGCAAAAAT |  |  |
| ChIP-qPCR |  | p50-800fw |  | TCCGAGGTAATGTGCTCTATGAAG |  |  |
| ChIP-qPCR |  | p50-800rv |  | ACAGACACCGGAGCAATACCC |  |  |
| ChIP-qPCR |  | p50-85fw |  | TACCGGCGACTCATTAAG |  |  |
| ChIP-qPCR |  | p50-85rv |  | TTGCGGAGTAAGGTTGAC |  |  |
| ChIP-qPCR |  | p73-998fw |  | CCCGTGCTGACATAGTTAGCG |  |  |
| ChIP-qPCR |  | p73-998rv |  | GGTACTGGGTCTGAACCACCAC |  |  |

^a^ MeDIP- / ChIP-qPCR: quantitative *real time* PCR analysis of MeDIP and ChIP samples, RT-qPCR: quantitative *real time* RT-PCR, BS: Bisulfite sequencing, COBRA: COBRA restriction analysis.

^b^ Fragment in Figure 3A which was amplified with this primer, if applicable
