# Supplementary material for: The Epigenetic Landscape of Latent Kaposi Sarcoma-Associated Herpesvirus Genomes
Source: PLoS Pathog. 2010 Jun 3;6(6):e1000935. doi: 10.1371/journal.ppat.1000935 (PMC2880564; doi:10.1371/journal.ppat.1000935)
Supplement: Protocol S1 — ChIP Protocol. (0.03 MB DOC) [file ppat.1000935.s002.doc]

**Protocol S1**

**Chromatin Immunoprecipitation Assay (ChIP)**

ChIP analysis was performed as described by Si et al. [1] and recommended by the array manufacturer (Agilent Mammalian ChIP-on-chip protocol V10.0, May 2008), with some modifications. For chromatin immunoprecipitation, protein from 5x10^6^ to 2x10^7^ cells was cross-linked to DNA with 1 % formaldehyde in PBS for 10 min (adherent cells) or 20 min (suspension cells) at room temperature. The reaction was quenched by adding 1/10^th^ volume 2.5 M glycine, cells were washed with PBS, scraped off the dish and collected. Cells were washed twice with ice cold PBS. All following steps were performed at 4°C. Lysis and wash buffers contained 1x protease inhibitor cocktail (Roche) and 1 mM Pefabloc® SC-Protease Inhibitor (Roth). Nuclei were isolated by incubation of cross-linked cells with 1 ml buffer I (50 mM Hepes-KOH, 140 mM NaCl, 1 mM EDTA, 10% glycerol, 0.5% NP-40, 0.25% Triton X-100) for 10 min on ice and pelleted by centrifugation (1,350 x g 5 min). The nuclei were subsequently washed with 1 ml buffer II (10 mM Tris-HCl, 200 mM NaCl, 1 mM EDTA, 0.5 mM EGTA), pelleted again and resuspended in 1 ml buffer III (1% SDS, 10 mM EDTA, 50 mM Tris-HCl). Chromatin was fragmented by sonication using a Bioruptor^TM^ (Diagenode) to an average length of 100-500 bp. After addition of 100 µl 10% Triton X-100, cell debris was pelleted by centrifugation (20,000 x g, 4°C) and supernatants were collected. For each individual IP, chromatin from 1x10^6^ cells in a maximum volume of 200µl was diluted with dilution buffer (0.01 % SDS, 1.1 % Triton X-100, 1.2 mM EDTA, 16.7 mM Tris-HCl, 167 mM NaCl) to a final volume of 2 ml. To reduce non-specific background, chromatin was pre-incubated with 60 µl salmon-sperm DNA protein-A agarose beads (Upstate). Antibodies (2 to 10 µg, depending on the antibody) specific for the histone modifications H3K9/K14-Ac (Upstate: #06-599), H3K4-me3 (Upstate: #04-745), H3K9-me3 (Upstate: #17-625) or H3K27-me3 (Upstate: #07-449) were added and incubated for 16 hrs at 4°C on a rotating wheel. 60 µl agarose beads were added to precipitate the chromatin-immunocomplexes for 1 hr at 4°C. Beads were washed once with low-salt buffer (0.1 % SDS, 1 % Triton X-100, 2 mM EDTA, 20 mM Tris-HCl, 150 mM NaCl), once with high-salt buffer (0.1 % SDS, 1 % Triton X-100, 2 mM EDTA, 20 mM Tris-HCl, 500 mM NaCl), once with LiCl-wash buffer (0.25 M LiCl, 1 % Nonidet P-40, 1 % Na-deoxycholate, 1 mM EDTA, 10 mM Tris-HCl) and twice with TE buffer. Chromatin was eluted from the beads in 210 µl elution-buffer (50 mM Tris-HCl pH 8.0, 10 mM EDTA, 1 % SDS) for 30 min at 65°C. 8 µl of a 5 M NaCl solution were added to 200 µl of the supernatant, and chromatin was de-crosslinked overnight at 65°C. Samples were diluted by adding 200 µl TE and RNA was degraded with 8 µl RNAseA (10 mg/ml) for 2 hrs at 37°C. For degradation of protein, 7 µl of CaCl_2_ solution (300 mM CaCl_2_ in 10 mM Tris-HCl) and 4 µl ProteinaseK (20 mg/ml Peqlab) were added and samples were incubated for 1h at 55°C. DNA was purified two times by phenol-chloroform extraction followed by a single chloroform extraction. DNA (400 µl in total) was precipitated with 1055 µl ethanol (100 %), 24 µl NaCl (5 M) and 3 µl glycogen (10 mg/ml) at -80°C for at least 30 min. After centrifugation (20.000 x g, 4°C, 15 min), pellets were washed once with ethanol (70 %) centrifuged again and dried in a vacuum centrifuge. DNA was resuspended in 70 µl of 10 mM Tris-HCl. For preparation of input controls, 1/4^th^ of the amount of chromatin that was used in the immunopreciptations was employed. Input samples were diluted in dilution buffer to a final volume of 200 µl and were treated identical to IP samples, starting with the decrosslinking step. Both samples were subsequenctly subjected to whole genome amplification and labeling using a linker mediated PCR protocol (Agilent Mammalian ChIP-on-chip protocol V10.0, May 2008), followed by microarray hybridization.

***References***

1. Si H, Verma SC, Robertson ES (2006) Proteomic analysis of the Kaposi's sarcoma-associated herpesvirus terminal repeat element binding proteins. J Virol 80: 9017-9030.
