# Supplementary material for: The Epigenetic Landscape of Latent Kaposi Sarcoma-Associated Herpesvirus Genomes
Source: PLoS Pathog. 2010 Jun 3;6(6):e1000935. doi: 10.1371/journal.ppat.1000935 (PMC2880564; doi:10.1371/journal.ppat.1000935)
Supplement: Protocol S2 — MeDIP Protocol. (0.04 MB DOC) [file ppat.1000935.s003.doc]

**Protocol S2**

**Methylated DNA Immunoprecipitation Assay (MeDIP)**

MeDIP analysis was essentially performed as described before [1,2,3,4] with some modifications. Highly pure genomic DNA serving as input for MeDIP was prepared by proteinase K digestion, followed by phenol chloroform extraction and ethanol precipitation. Negative and positive controls were prepared by mixing genomic DNA from KSHV-negative SLK cells with unmethylated or *in vitro* methylated KSHV bacmid DNA [5], respectively. The ratio of viral vs. cellular DNA was selected such that it mimics the episome content typically seen in KSHV-infected PEL cell lines and the SLKp line (approx. 30-40 copies per cell). The control DNA or DNA samples from KSHV-infected cells were resuspended in TE containing RNAseA and incubated for at least 2 hrs at 37°C to degrade RNA. DNA was sonicated to an average fragment size of 100-500 bp using a Bioruptor^TM^ (Diagenode). In order to allow quantification and normalization of the data, 0.2 ng of *in vitro* methylated pCR2.1 plasmid were added to 5 µg of sheared DNA and diluted in TE to a final volume of 250 µl. 50 µl (1 µg) of the dilution were saved as an input control, and another 250 µl TE were added to the remaining 200 µl. Denaturing of DNA was performed by incubation at 98°C for 10 min, followed by incubation on ice for 10 min. Subsequently, 51 µl 10x IP-buffer (100 mM Na-phosphate buffer, pH 7.0, 1.4 mM NaCl, 0.5 % Triton X-100) were added. Immunoprecipitation was performed by addition of 2.5 µg of a 5´-methylcytidine specific antibody (MAb-5MECYT-100, Diagenode) and incubation for 2 hrs on a rotating wheel at 4°C. 50 µl Dynabeads® M-280 Sheep anti-Mouse IgG (Invitrogen) were washed once with 0.1 % BSA in PBS, resuspended in 50 µl 1x IP-buffer, added to the DNA-antibody complexes and incubated for 2 hrs. Samples were washed three times with 1x IP-buffer for 10 min. Beads were resuspended in 150 µl elution buffer (50 mM Tris-HCl pH 8.0, 10 mM EDTA, 0.5 % SDS) and 3.5 µl proteinase K were added. Samples were incubated shaking at 55°C for 15 min followed by 2 min at 65°C. Eluate was collected, elution was repeated once and the eluates were combined. DNA was purified twice by phenol-chloroform extraction, followed by a single round of chloroform extraction. DNA (300 µl in total) was precipitated with 810 µl ethanol (100 %), 24 µl NaCl (5 M) and 3 µl glycogen (10 mg/ml) at -80°C for at least 30 min. After centrifugation (20.000 x g, 4°C, 15 min), pellets were washed once with ethanol (70 %) centrifuged again, dried in a speedvac and resuspended in 15.5 µl H_2_O. Input controls were prepared by adding 250 µl water to 50 µl of the sheared DNA input samples and treated identical to the IP samples, starting with the ethanol precipitation step. The samples were subsequently analyzed by qPCR and/or microarray hybridization.

***References***

1. Weber M, Davies JJ, Wittig D, Oakeley EJ, Haase M, et al. (2005) Chromosome-wide and promoter-specific analyses identify sites of differential DNA methylation in normal and transformed human cells. Nat Genet 37: 853-862.

2. Weber M, Hellmann I, Stadler MB, Ramos L, Paabo S, et al. (2007) Distribution, silencing potential and evolutionary impact of promoter DNA methylation in the human genome. Nat Genet 39: 457-466.

3. Zilberman D, Gehring M, Tran RK, Ballinger T, Henikoff S (2007) Genome-wide analysis of Arabidopsis thaliana DNA methylation uncovers an interdependence between methylation and transcription. Nat Genet 39: 61-69.

4. Reynaud C, Bruno C, Boullanger P, Grange J, Barbesti S, et al. (1992) Monitoring of urinary excretion of modified nucleosides in cancer patients using a set of six monoclonal antibodies. Cancer Lett 61: 255-262.

5. Zhou FC, Zhang YJ, Deng JH, Wang XP, Pan HY, et al. (2002) Efficient infection by a recombinant Kaposi's sarcoma-associated herpesvirus cloned in a bacterial artificial chromosome: application for genetic analysis. J Virol 76: 6185-6196.
