# Supplementary figures and images for: The Epigenetic Landscape of Latent Kaposi Sarcoma-Associated Herpesvirus Genomes
Source: PLoS Pathog. 2010 Jun 3;6(6):e1000935. doi: 10.1371/journal.ppat.1000935 (PMC2880564; doi:10.1371/journal.ppat.1000935)

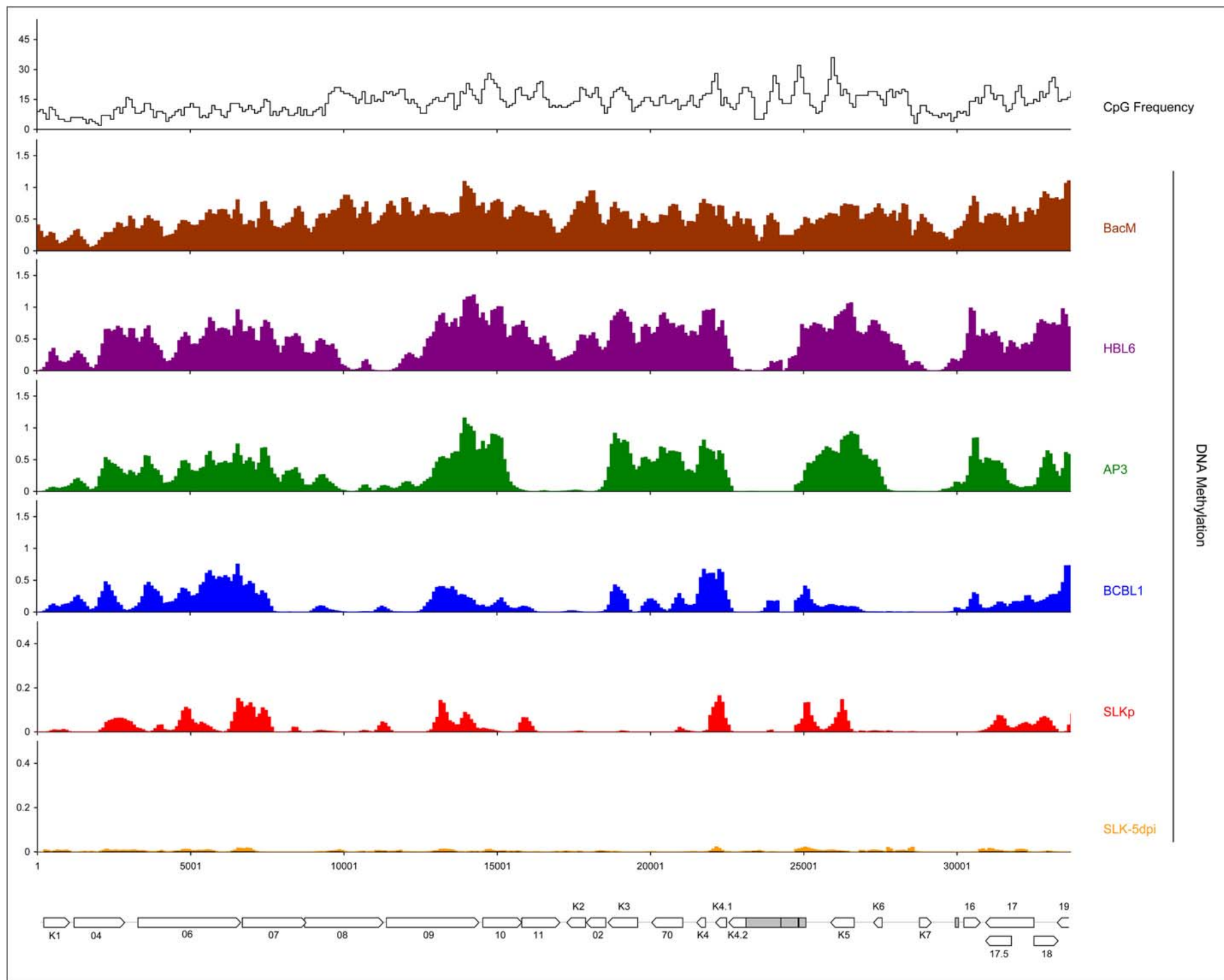

Figure S1

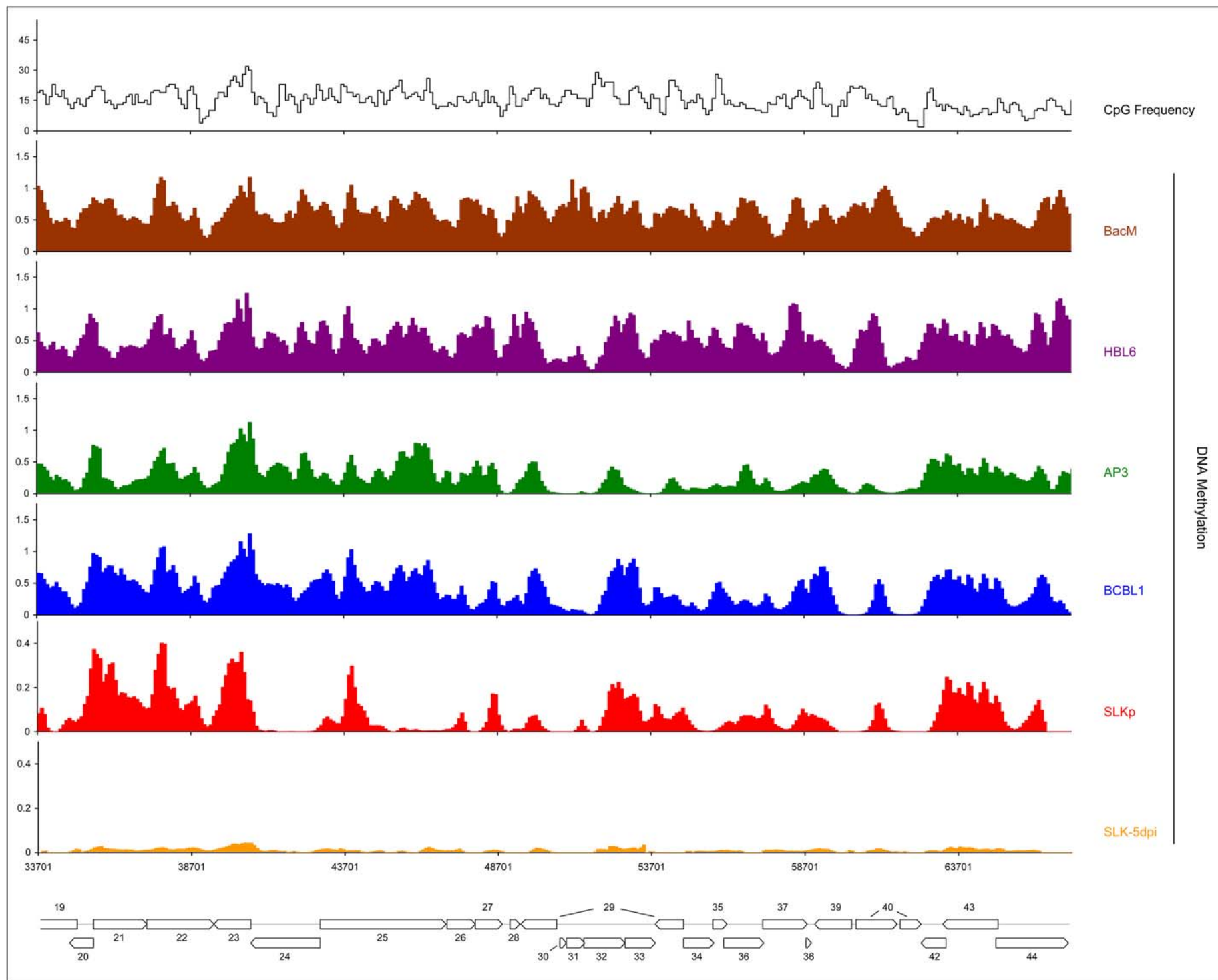

Figure S1, contn'd

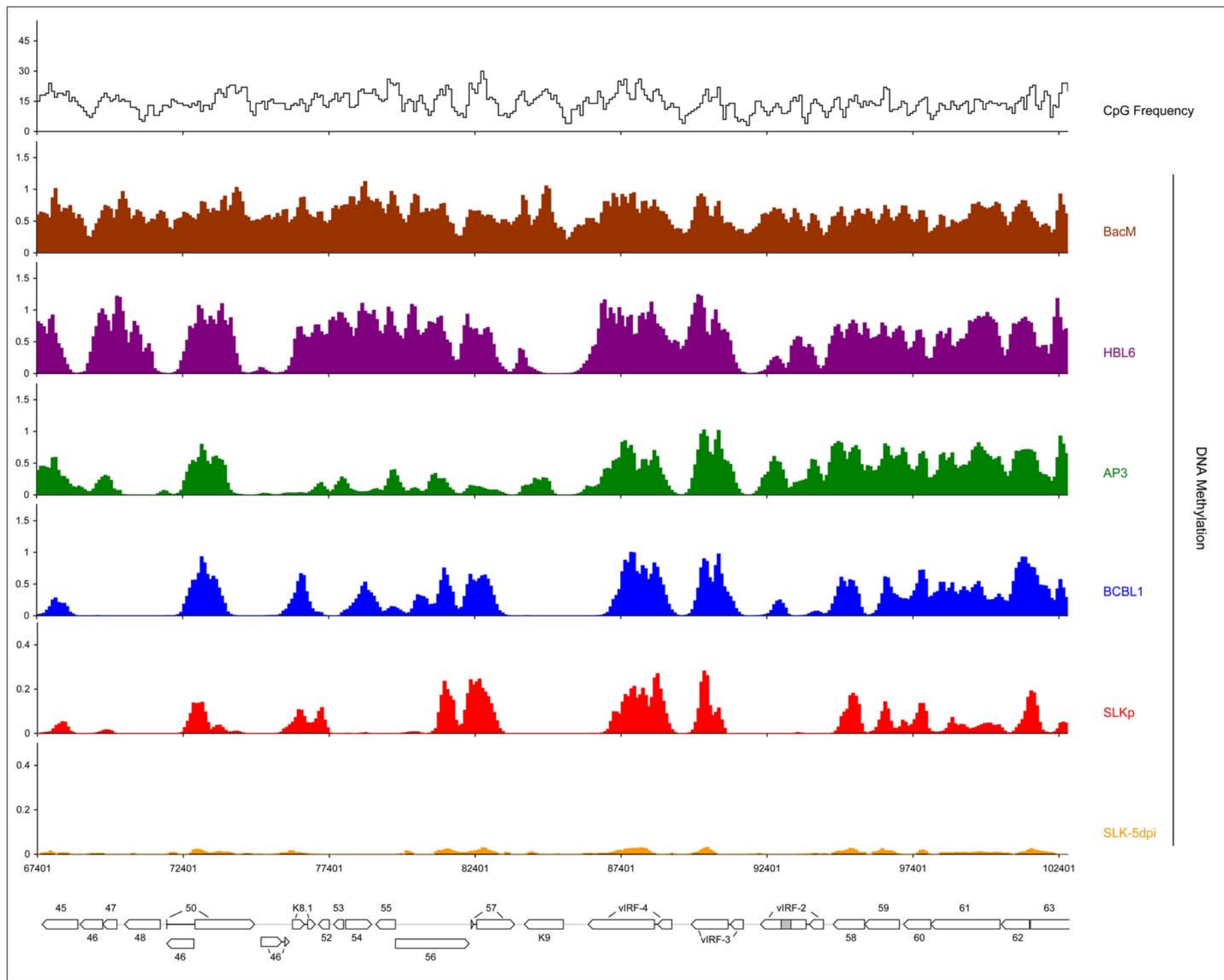

Figure S1, contn'd

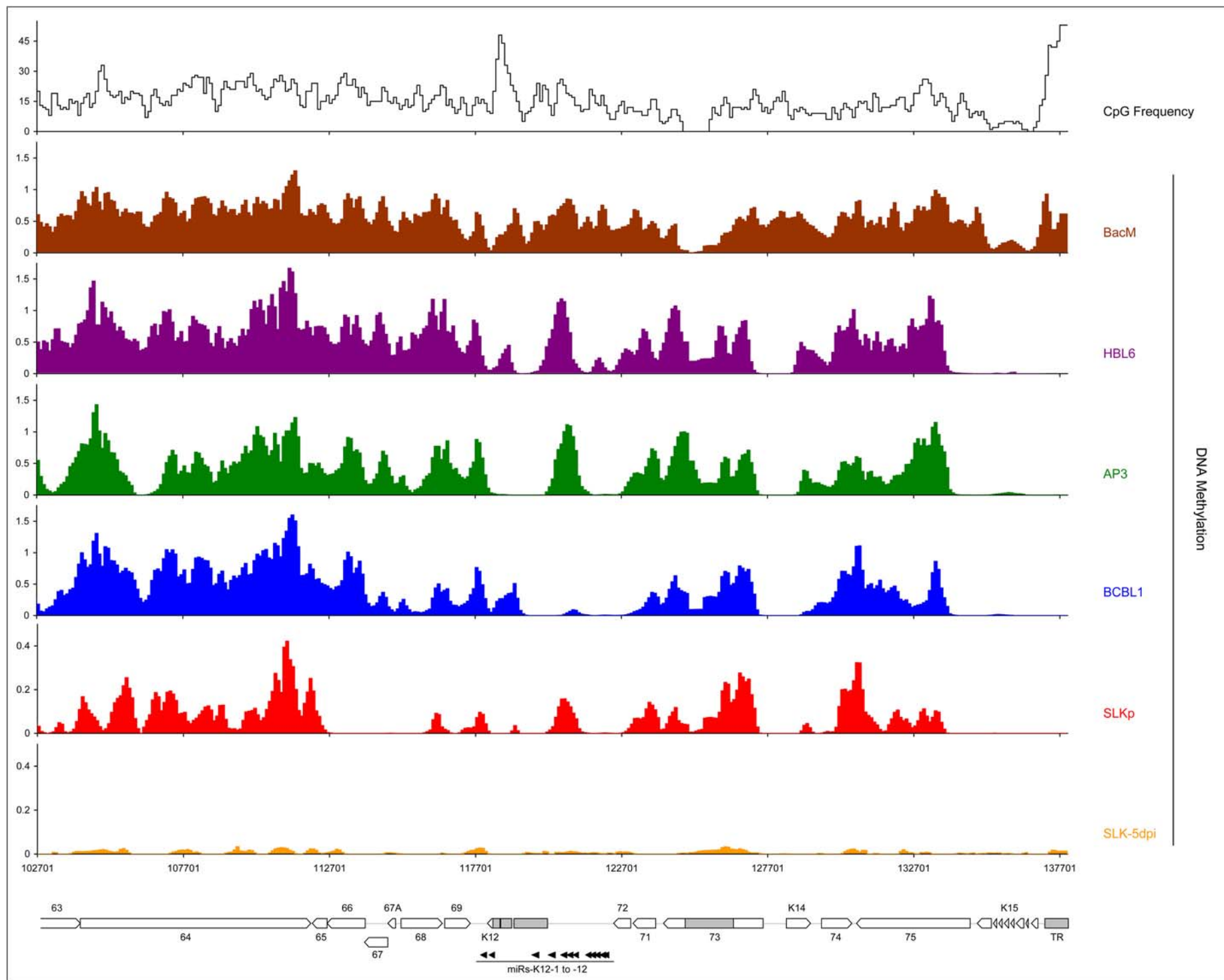

Figure S1, contn'd

Supplement: Figure S1 — Global DNA methylation patterns of latent KSHV genomes (Higher magnification of data presented in Figure 2). (0.50 MB PDF) [file ppat.1000935.s005.pdf]

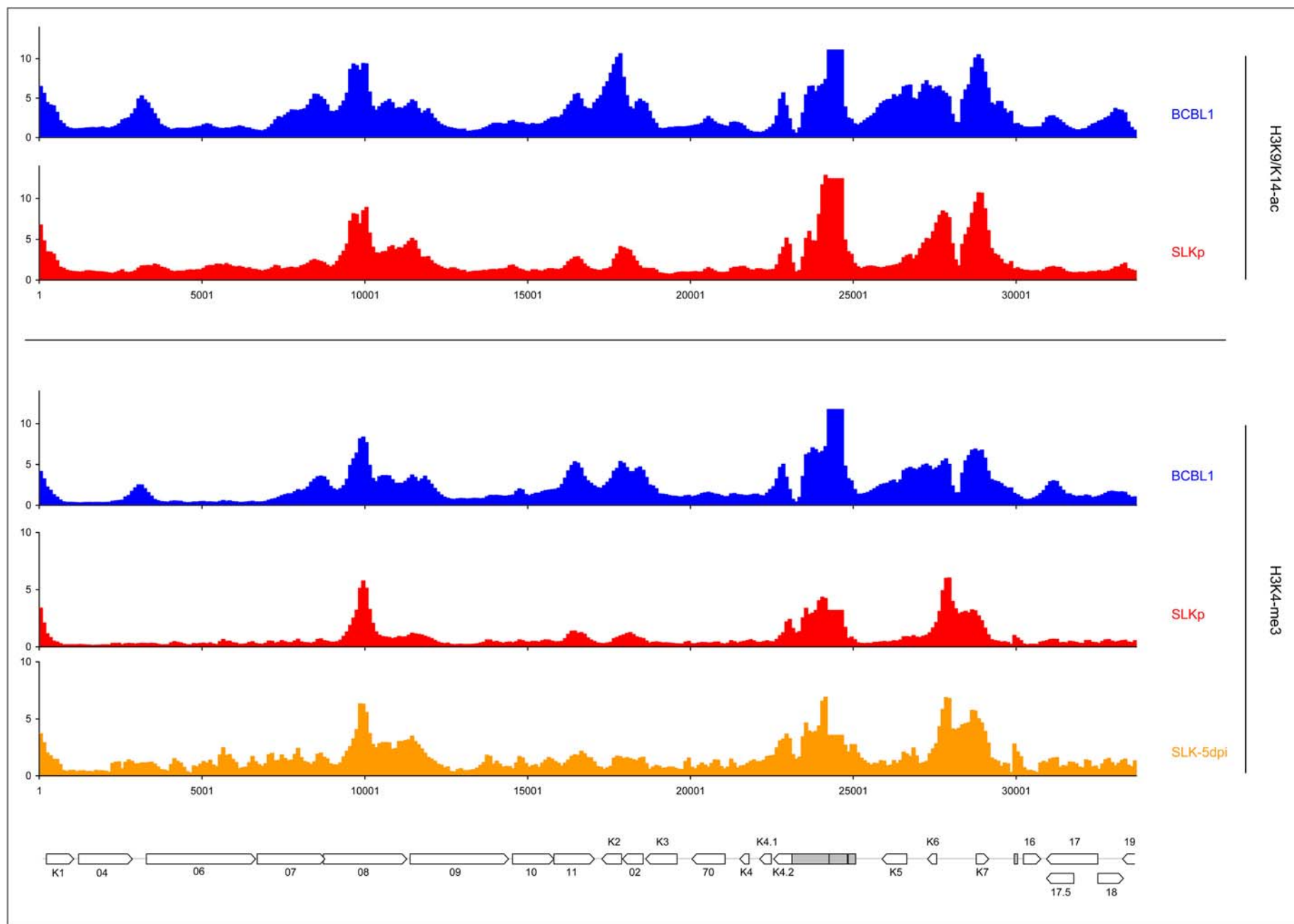

Figure S2

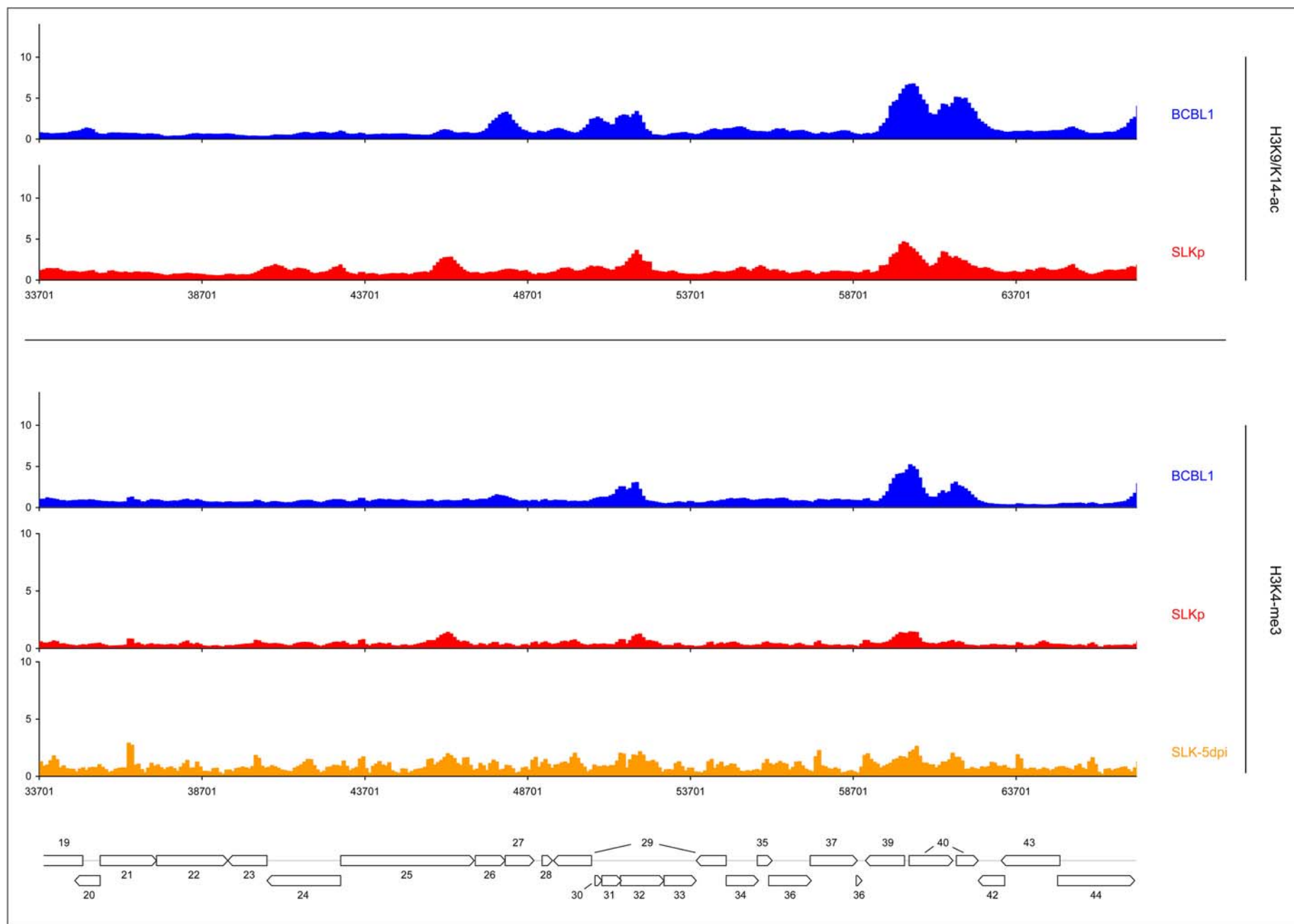

Figure S2, contn'd

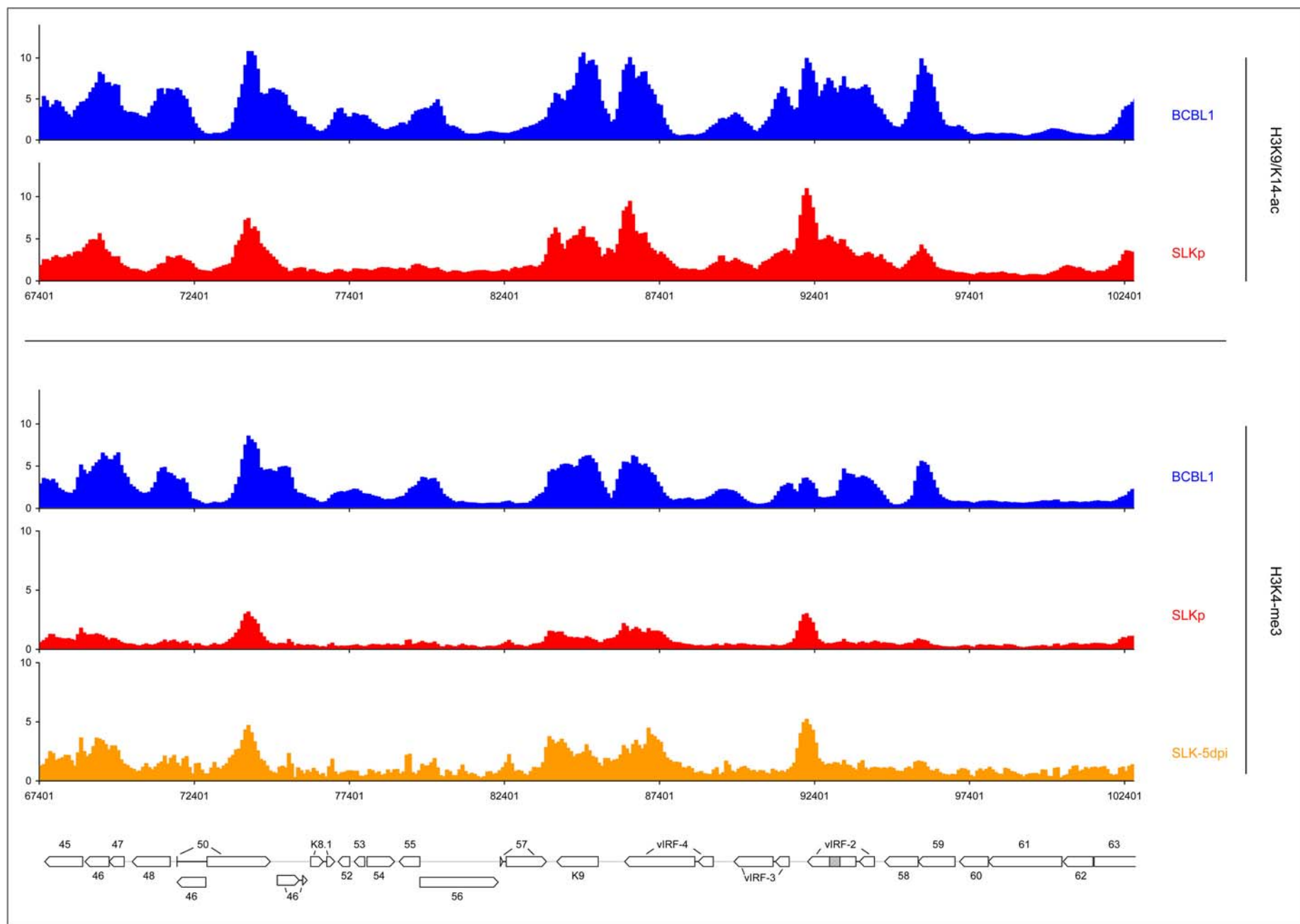

Figure S2, contn'd

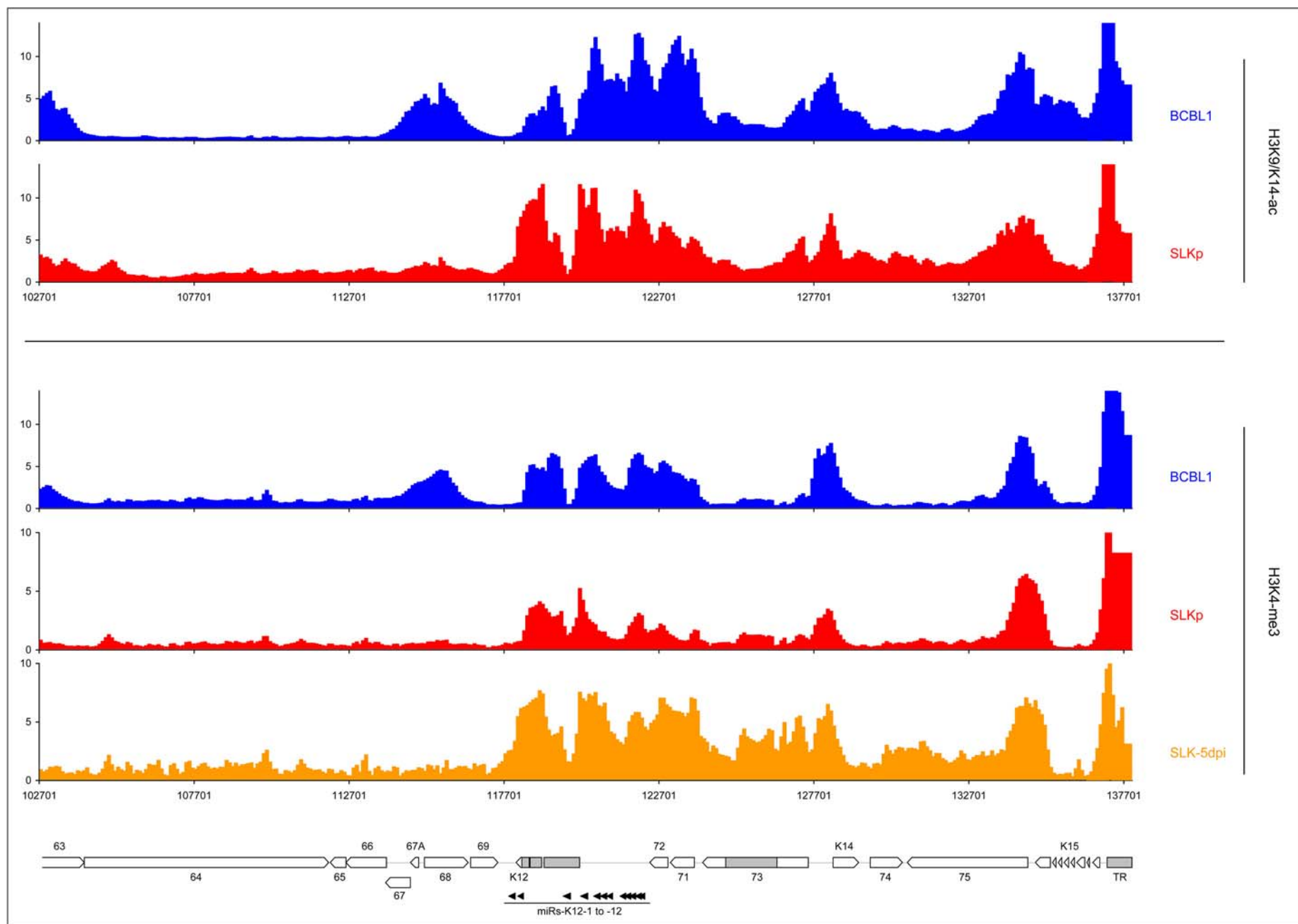

Figure S2, contn'd

Supplement: Figure S2 — Global patterns of H3K9/K14 Acetylation and H3K4 tri-methylation on latent KSHV genomes (Higher magnification of data presented in Figure 6). (0.41 MB PDF) [file ppat.1000935.s006.pdf]

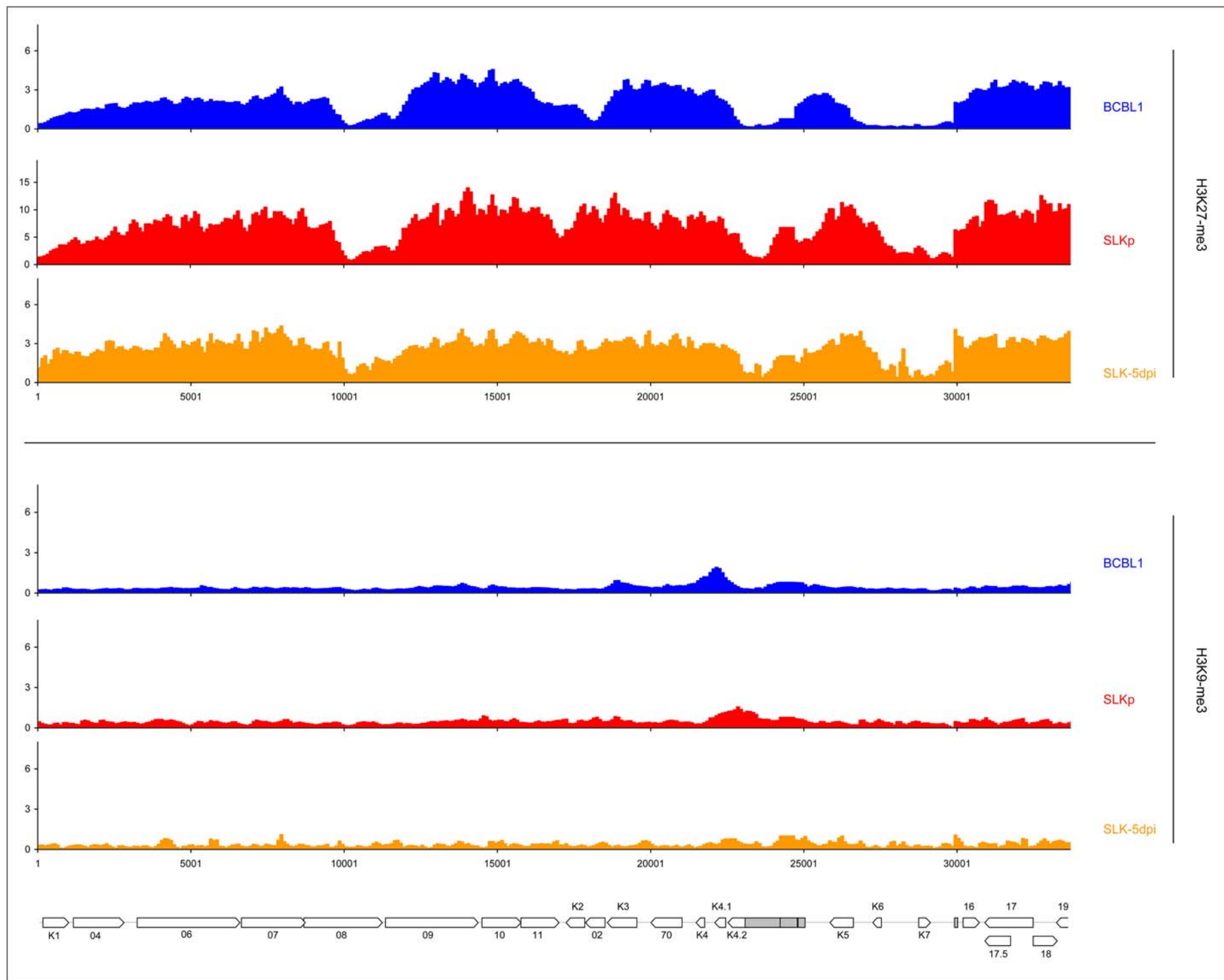

Figure S3

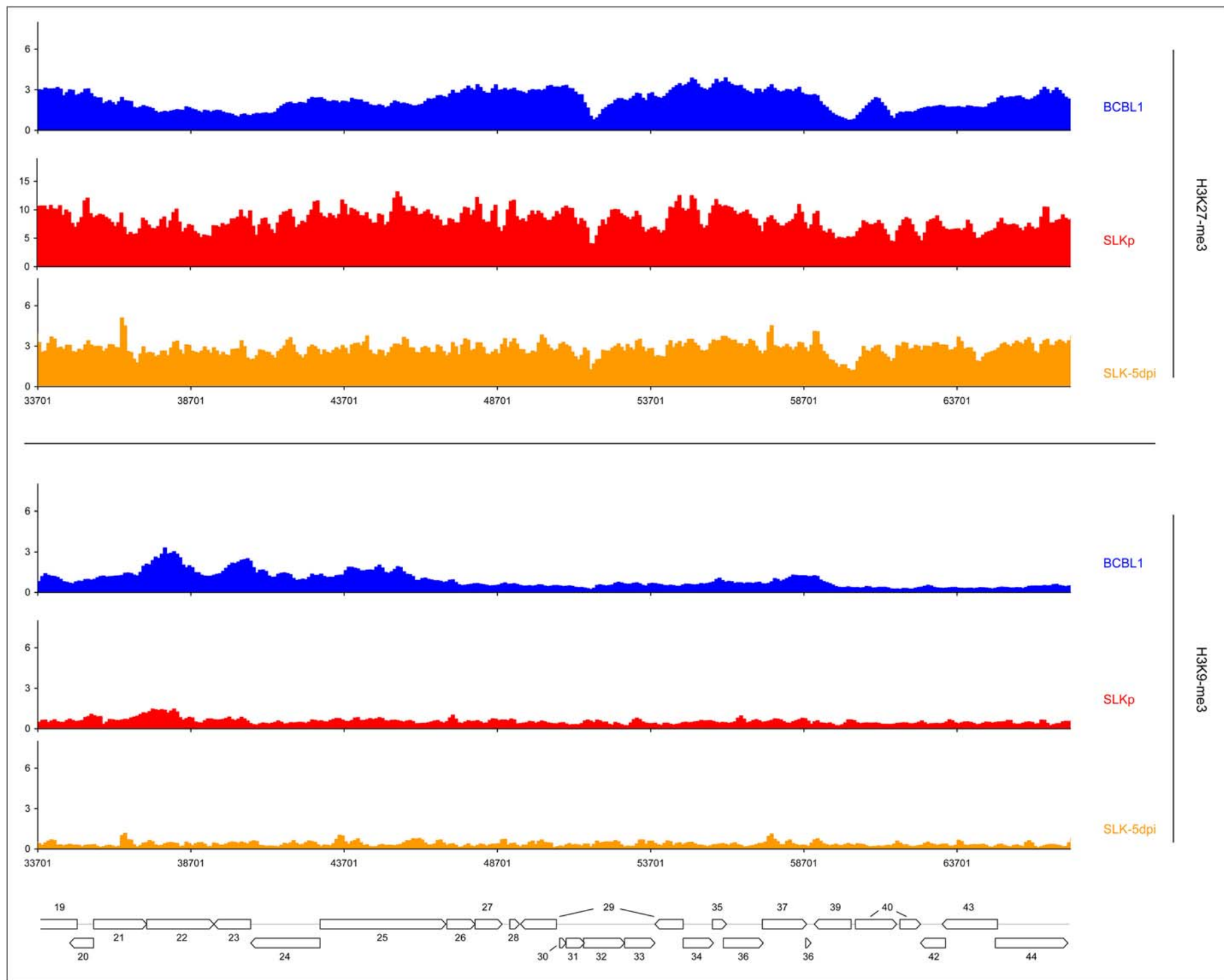

Figure S3, contn'd

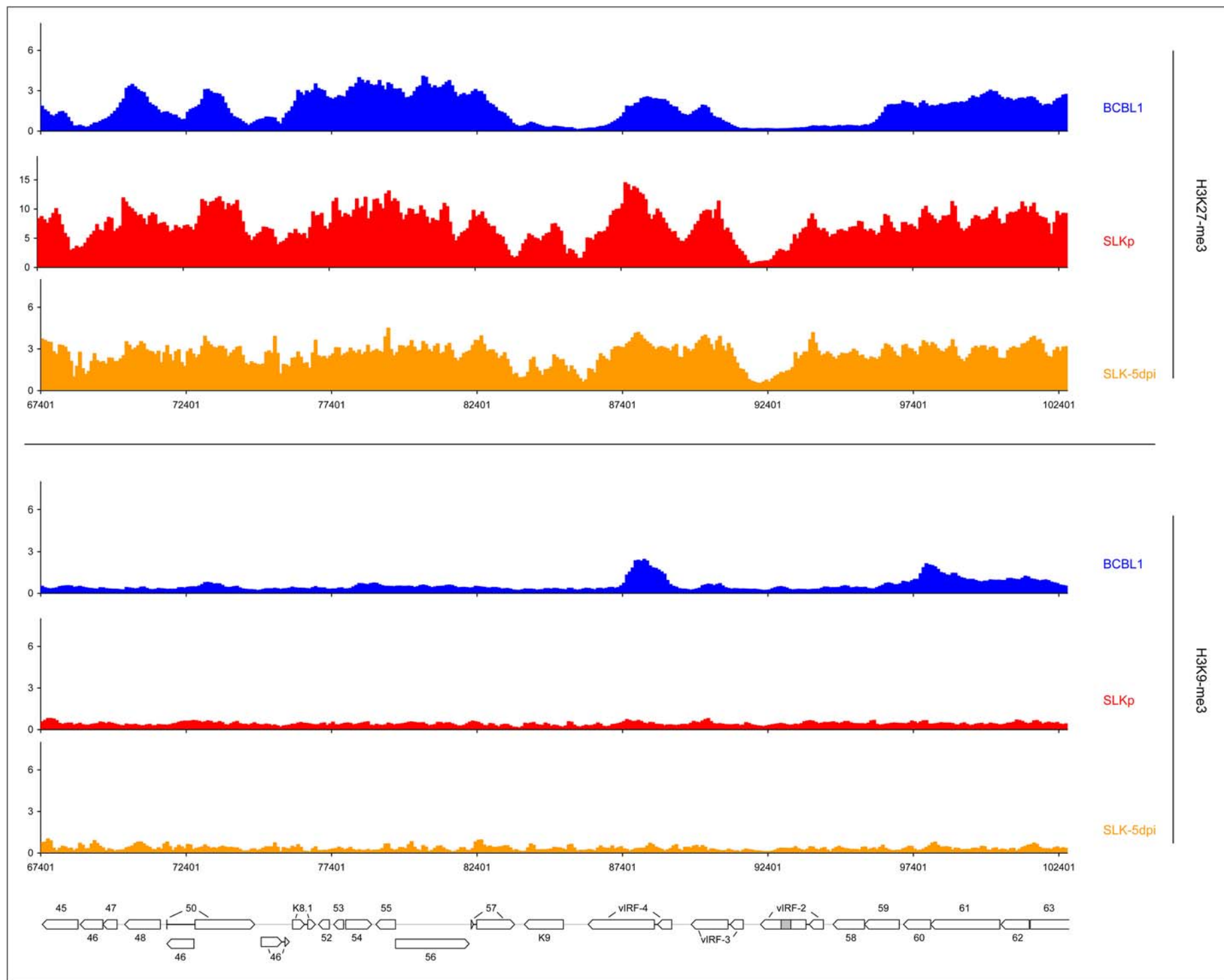

Figure S3, contn'd

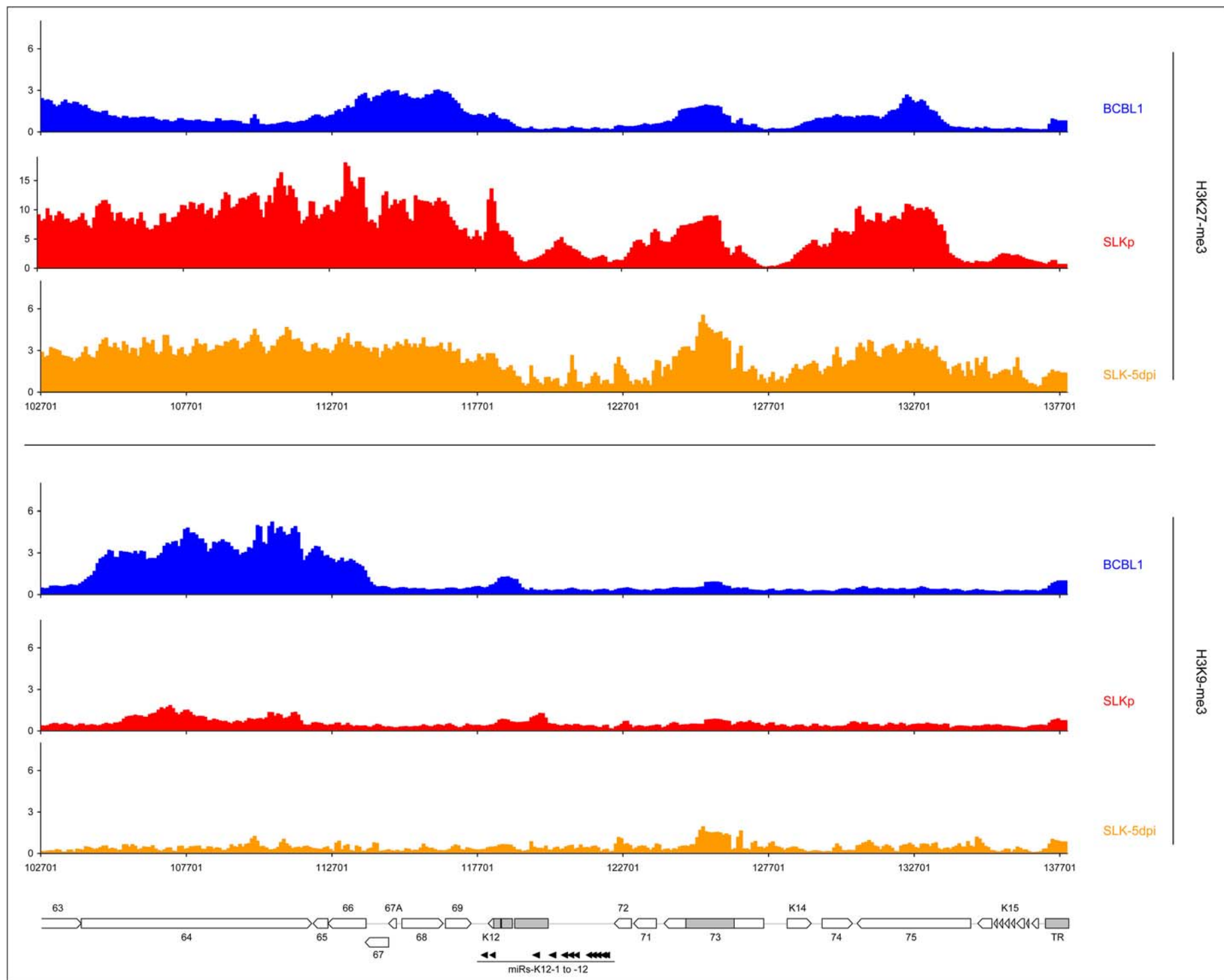

Figure S3, contn'd

Supplement: Figure S3 — Global patterns of H3K27 and H3K9 tri-methylation on latent KSHV genomes (Higher magnification of data presented in Figure 7). (0.44 MB PDF) [file ppat.1000935.s007.pdf]

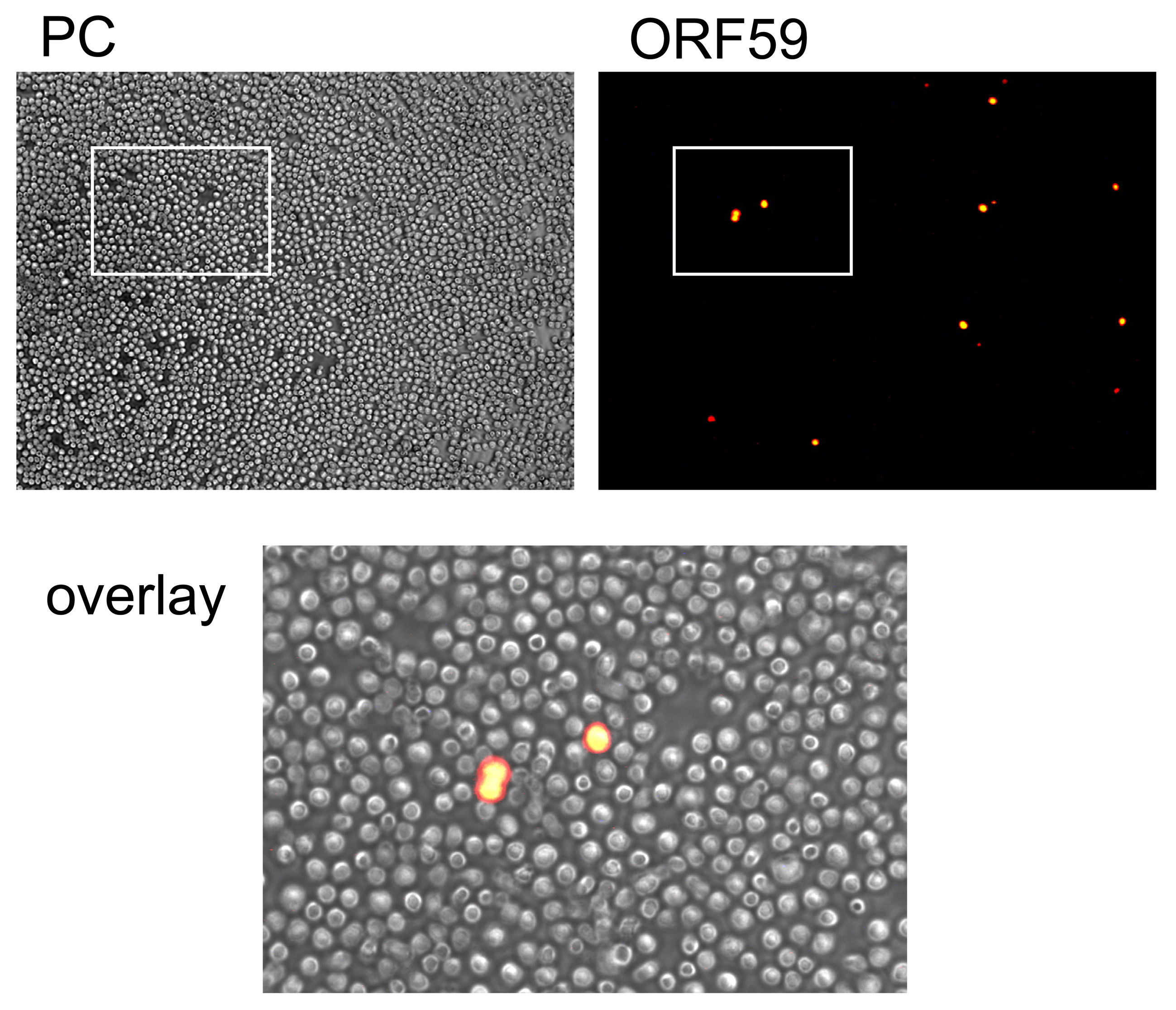

Supplement: Figure S4 — Spontaneous reactivation in BCBL1 cells. BCBL1 cells were analyzed by immunofluorescence for the expression of the ORF59 gene product. A phase contrast image (PC) is shown to the left, and an enlarged overlay of the section framed by the white rectangle is shown at the bottom. (3.17 MB TIF) [file ppat.1000935.s008.tif]

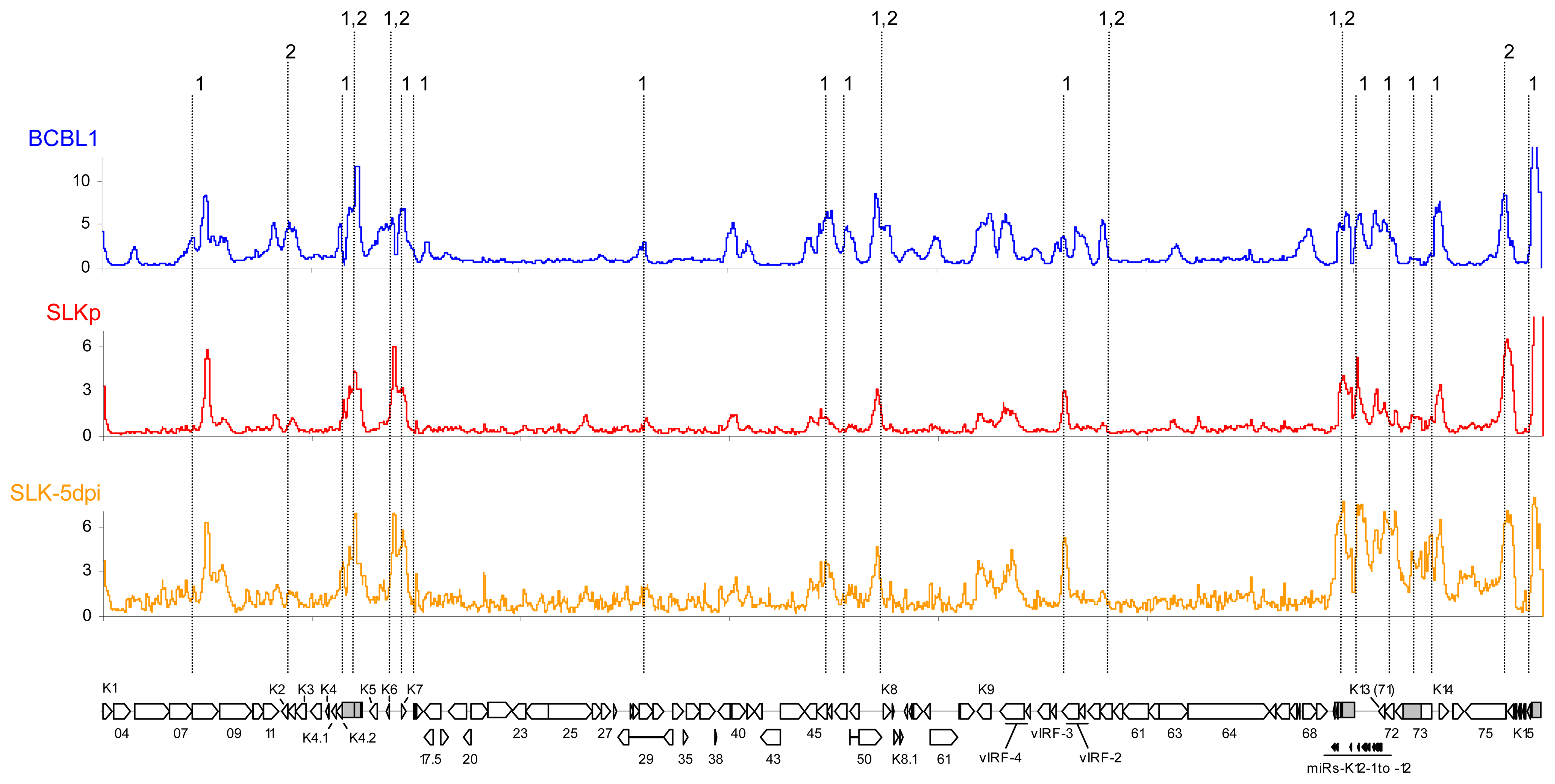

Supplement: Figure S5 — Rta binding sites and global patterns of H3K4-me3 on latent KSHV genomes. H3K4 tri-methylation (H3K4-me3) patterns in BCBL1, SLKp cells as well as SLK cultures at 5 days post infection (SLK-5dpi) are shown as described in the legend to Figure 6. The location of regions which were found to harbor Rta binding sites in genome-wide screens performed by Chen et al. [48] or Ellison et al. [24] is indicated by dotted lines. The labeling above the lines indicates whether these regions were identified by Chen et al. (labelled “1”), Ellison et al. (“2”), or in both studies (“1,2”). (1.02 MB TIF) [file ppat.1000935.s009.tif]
